# Meta-Analysis of the Immunogenicity and Tolerability of Pandemic Influenza A 2009 (H1N1) Vaccines

**DOI:** 10.1371/journal.pone.0024384

**Published:** 2011-09-06

**Authors:** Lamberto Manzoli, Corrado De Vito, Georgia Salanti, Maddalena D'Addario, Paolo Villari, John P.A. Ioannidis

**Affiliations:** 1 Section of Hygiene, Epidemiology, Pharmacology and Legal Medicine, University “G. d'Annunzio” of Chieti, Chieti, Italy; 2 Department of Public Health and Infectious Diseases, Sapienza University of Rome, Rome, Italy; 3 Department of Hygiene and Epidemiology, University of Ioannina School of Medicine, Ioannina, Greece; 4 Stanford Prevention Research Center, Department of Medicine and Department of Health Research and Policy, Stanford University School of Medicine, Stanford, California, United States of America; University of Hong Kong, Hong Kong

## Abstract

**Background:**

Although the 2009 (H1N1) influenza pandemic officially ended in August 2010, the virus will probably circulate in future years. Several types of H1N1 vaccines have been tested including various dosages and adjuvants, and meta-analysis is needed to identify the best formulation.

**Methods:**

We searched MEDLINE, EMBASE, and nine clinical trial registries to April 2011, in any language for randomized clinical trials (RCTs) on healthy children, adolescents, adults and the elderly. Primary outcome was the seroconversion rate according to hemagglutinination-inhibition (HI); secondary outcomes were adverse events. For the primary outcome, we used head-to-head meta-analysis and multiple-treatments meta-analysis.

**Results:**

Eighteen RCTs could be included in all primary analyses, for a total of 76 arms (16,725 subjects). After 2 doses, all 2009 H1N1 split/subunit inactivated vaccines were highly immunogenic and overcome CPMP seroconversion criteria. After 1 dose only, all split/subunit vaccines induced a satisfactory immunogenicity (> = 70%) in adults and adolescents, while only some formulations showed acceptable results for children and elderly (non-adjuvanted at high-doses and oil-in-water adjuvanted vaccines). Vaccines with oil-in-water adjuvants were more immunogenic than both nonadjuvanted and aluminum-adjuvanted vaccines at equal doses and their immunogenicity at doses < = 6 µg (even with as little as 1.875 µg of hemagglutinin antigen) was not significantly lower than that achieved after higher doses. Finally, the rate of serious vaccine-related adverse events was low for all 2009 H1N1 vaccines (3 cases, resolved in 10 days, out of 22826 vaccinated subjects). However, mild to moderate adverse reactions were more (and very) frequent for oil-in-water adjuvanted vaccines.

**Conclusions:**

Several one-dose formulations might be valid for future vaccines, but 2 doses may be needed for children, especially if a low-dose non-adjuvanted vaccine is used. Given that 15 RCTs were sponsored by vaccine manufacturers, future trials sponsored by non-industry agencies and comparing vaccines using different types of adjuvants are needed.

## Introduction

According to WHO statistics, from April 2009 to August 2010 more than 214 countries reported laboratory confirmed cases of pandemic influenza 2009 H1N1, with over 18,449 deaths [1]. Vaccination typically represents the major tool to reduce the morbidity, mortality and economic effects of influenza pandemics [2], [3], and extraordinary efforts were devoted to the development and administration of 2009 H1N1 vaccines worldwide [4]. Although the pandemic officially ended in August 2010 [5], the virus was detected in 13.4% of the specimens tested in USA during 2010–11 season [6], and WHO accordingly recommended that an A/California/7/2009 (H1N1)-like virus be used for influenza vaccines in the 2010–11 season [7]. In fact, it is likely that the same virus will continue to cause epidemics and pandemics in future years.

Several studies have assessed the immunogenicity and safety of various formulations of 2009 H1N1 vaccine, comparing a wide variety of doses with or without different adjuvants [8], [9], [10], [11], [12], [13], [14], [15], [16], [17], [18], [19], [20], [21], [22], [23], [24], [25], [26], [27], [28], [29], [30], [31], [32], [33], [34], [35], [36], [37], [38], [39]. Although no major harms were noted for any of the vaccine formulations, the immunogenicity varied and several issues remain about the relative merits of each formulation that are very difficult to address examining single trials. Specifically, there are several outstanding questions: how do the seroconversion rates, at various ages, compare for diverse vaccine formulations? Which is the lowest dose inducing a satisfactory immunogenicity for adjuvanted and non-adjuvanted formulations? Does the second dose relevantly increase the immunogenicity of the various vaccine formulations (and in what ages)? Are adjuvanted 2009 H1N1 vaccines more immunogenic than non-adjuvanted vaccines at equal doses of hemagglutinin antigen (HA)? Among the adjuvants, is aluminum more/less immunogenic than other oil-in-water adjuvants (i.e. MF-59, AS03) at equal dosages? To help answer these questions, we performed a meta-analysis of all clinical trials on H1N1 vaccines. We used both traditional meta-analysis and multiple treatment meta-analysis (MTM) [40], [41] to combine data across a large number of diverse comparisons of different formulations. We also systematically reviewed the available evidence on the relative safety and harms of different vaccine formulations.

## Methods

### Research in Context: Search Strategy and Quality Assessment

Trials evaluating influenza 2009 H1N1 vaccine safety and immunogenicity in healthy humans that were not previously vaccinated with 2009 H1N1 vaccines were retrieved through a search in MEDLINE (limited to publications after the year 2008); EMBASE; and several clinical trial registries (Cochrane Controlled Clinical Trial Register, ISRCTN, US ClinicalTrials.gov, WHO ICTRP, GSK Clinical Study Register, and Indian, Australian New Zealand and Chinese Clinical Trial Registries), with no language restriction (last update April 1, 2011). Search terms were “influenza”, “vaccine OR vaccines OR vaccination”, and “H1N1 or pandemic” in all fields. The bibliographies of all relevant articles including reviews were reviewed for further references. We aimed to identify both randomized controlled trials (RCTs) as well as studies where a single vaccine formulation was tested or individuals were non-randomly allocated. Only the randomized controlled trials (RCTs) were used in the primary analyses (see below). When it was not possible to extract some outcomes from a study, the corresponding author was contacted.

Data extraction and quality assessment were made independently by two reviewers. We assessed aspects of the reported methodological quality of each RCT pertaining to randomization (generation of allocation sequences and concealment of allocation), blinding, and adequacy of analyses (including dropouts/withdrawals) [42].

### Primary Outcome: Immunogenicity

Typical outcomes for the evaluation of influenza vaccine immunogenicity are seroconversion (the proportion of subjects with a pre-vaccination hemagglutinination-inhibition – HI – antibody titer < = 1∶10 and a 3–4 week post-vaccination titer > = 1∶40, or a pre-vaccination titer > = 1∶10 and an increase in the titer by a factor of four or more after vaccination), and seroresponse/seroprotection (the proportion of subjects with post-vaccination HI titers > =  1∶40) [43]. When both seroconversion and seroresponse data were available in a study, only seroconversion data were used, as they may be less sensitive to baseline status (seroresponse rates also include subjects with pre-vaccination immunity to H1N1). Importantly, immunogenicity is considered a good surrogate of the truly important clinical outcome of clinical protection. It is has been observed that seroconversion with a title of 1∶40 corresponds to 50% clinical protection and a meta-analysis shows that there is a relationship between the exact titre and the proportion of clinical protection [44]. However, we did not have sufficient data to examine separate titre thresholds in this meta-analysis.

### Secondary Outcomes: Harms

We considered the main adverse events (AEs) recorded in the setting of influenza immunization: fever, any systemic reaction, injection-site pain and any local reaction. AEs are usually self-reported during the first 7–10 days after vaccination. When a study reported data on harms at further time intervals, only the data regarding the first 7–10 days were used to ensure comparability. AEs definition was not always fully standardized and similar across trials: in case of multiple definitions, we used the one closer to the guidelines proposed by the Committee for Proprietary Medicinal Products (CPMP) [45]. We separately recorded serious events (life-threatening events, or events resulting in persistent disability, hospitalization or death). Since many studies reported mild and moderate adverse events together, these were analyzed as a single group. We extracted separately the adverse event rates after the first and second dose. For the computation of the AE rate after both doses, if a study only provided data separately by dose, we extracted the highest event rate [43].

### Data Analysis

The main analyses for the primary outcome used only data from the RCTs and combination of the data respected the randomization. First, we performed random-effect meta-analyses comparing the various formulations in head-to-head comparisons [46]. The following meta-analyses were made: higher versus immediately lower dose (overall and separately for 30 µg vs 15 µg, and 15 µg vs 7.5 µg) for non-adjuvanted vaccines; higher versus immediately lower dose (overall and separately for 15 µg vs 7.5 µg, and 7.5 µg vs 1.88–5.25 µg) for adjuvanted vaccines. Moreover, for 7.5 µg and 15 µg doses, we performed a comparative evaluation between non-adjuvanted and adjuvanted vaccines (overall, by age and by adjuvant type). The results were expressed with risk ratio (RR) and 95% confidence interval (95% CI). The Mantel-Haenszel method (fixed-effects model) [47] was also for comparison with random effect calculations. Statistical heterogeneity was quantified using the I^2^ metric [48]; in case of limited data I^2^ metric 95% confidence intervals are typically large [49] and thus inferences on the magnitude of the statistical heterogeneity should be cautious.

Second, we synthesized the evidence from the entire network of comparisons of all treatments (including placebo) using Multiple-Treatments Meta-analysis [40], [50]. The methodology combines direct and indirect estimates and fitted within a Bayesian framework estimates all relative effect sizes, their credibility intervals that can be used to rank the treatments [51]. MTM has the advantage that it can incorporate the evidence from all comparisons of different treatments within a single analysis. This allows a better appreciation of the relative merits of each treatment within a common analytical framework. Moreover, for each comparison of two specific treatments, the available evidence is more than what is used in the simple head-to-head direct comparison meta-analysis, because we also gain information from how each treatment has performed against other treatments outside the pair of interest through indirect comparisons [52]. An important assumption underlying the model is that evidence is consistent, i.e. direct and indirect evidence are in agreement. The assumption of consistency can be tested by comparing the consistency and inconsistency models with respect to the trade-off between model fit and parsimony [53]. We used the odds ratio (OR) as the metric of choice for running the MTM calculations, since it is a symmetric measure and may tend to minimize heterogeneity when data are combined across diverse dosing regimens. The posterior ORs have been translated also to risk differences (RDs) against a baseline dosing regimen (1.88–5.25 µg with non-aluminum adjuvants) so as to allow a more direct clinical interpretation of the data. In the MTM framework, according to the available data we considered the following formulations as separate nodes in the network: placebo; doses 1.88–5.25 µg; 1.88–5.25 µg +Aluminum; 1.88–5.25 µg + other adjuvants; 7.5 µg, 7.5 µg + Aluminum; 7.5 µg + other adjuvants; 15 µg; 15 µg + Aluminum; 15 µg + other adjuvants (for dose one only); 21–30 µg; therefore having eleven different interventions to evaluate after one or single dose; ten after two doses.

For each of the harms outcomes, given the suboptimal standardization of definitions and missingness of data, inconsistency would be likely to occur, thus we did not perform any MTM calculations. We only used meta-analysis of direct randomized comparisons.

As sensitivity analysis, we also performed meta-analysis of proportions combining the data on single arms. This sensitivity analysis has the advantage that it allowed also the inclusion of data from non-randomized studies. Given that these analyses do not respect the randomization, even for data coming from RCTs, they should be seen with extra caution.

In order to investigate the additional immunogenicity provided by two doses of vaccine as opposed to a single dose, all analyses were made twice: the first time including the results after one dose only; the second time containing the results after both doses. To probe onto the benefits of two administrations vs one, we also performed additional proportion meta-analyses in which we summarized, for each formulation, the absolute percentages of subjects who achieved seroconversion/seroresponse only after the second dose. As an example, if a trial reported 70% of seroconversions after the first dose, and 80% after the second dose, the absolute percentage of subjects who benefited from the second dose was 10%.

To try to investigate the potential role of methodological quality on summary estimates, we stratified studies into two classes: higher and lower risk of bias. A RCT was considered at lower risk of bias if the procedures of allocation concealment were appropriately reported, and at least one of the following processes was adequately described (and correctly made): random sequence generation and handling of dropouts/withdrawals. Blinding as well as other classifications were also considered but they were almost overlapping (most non blinded studies were also at higher risk of bias) and did not result in any substantial change in the analyses. A non randomized trial was considered at lower risk of bias if the procedures to handle withdrawals and dropouts (and their numbers and reasons for) were correctly described. We only stratified proportion meta-analyses by quality because, due to the unavoidable age-stratification, especially in the most interesting comparison (adjuvanted versus non-adjuvanted vaccines), very few or even no lower risk of bias study could be found in each cell of the various head-to-head meta-analyses and into the multiple-treatment meta-analyses.

Finally, direct comparison meta-analyses were stratified by age-class (children: from 6 months to 9 years; adolescents: from 10 to 17 years; adults: from 18 to 64 years; elderly: from 65 years). We performed the MTM computations considering data from all age-classes to avoid overfragmentation of the data in a complex network that has many nodes.

We used StatsDirect 2.7.8 (StatsDirect Ltd, Altrincham, UK, 2010), RevMan 5.0 (Copenhagen: The Nordic Cochrane Centre, The Cochrane Collaboration, 2008), and WinBUGS (WinBUGS User Manual Version 1.4.3. Available at: http://www.mrc-bsu.cam.ac.uk/bugs) to perform proportion meta-analyses, direct comparison meta-analyses, and MTM, respectively. The protocol of the review is available online as supporting information.

## Results

### Characteristics of eligible studies

**Figure 1:**
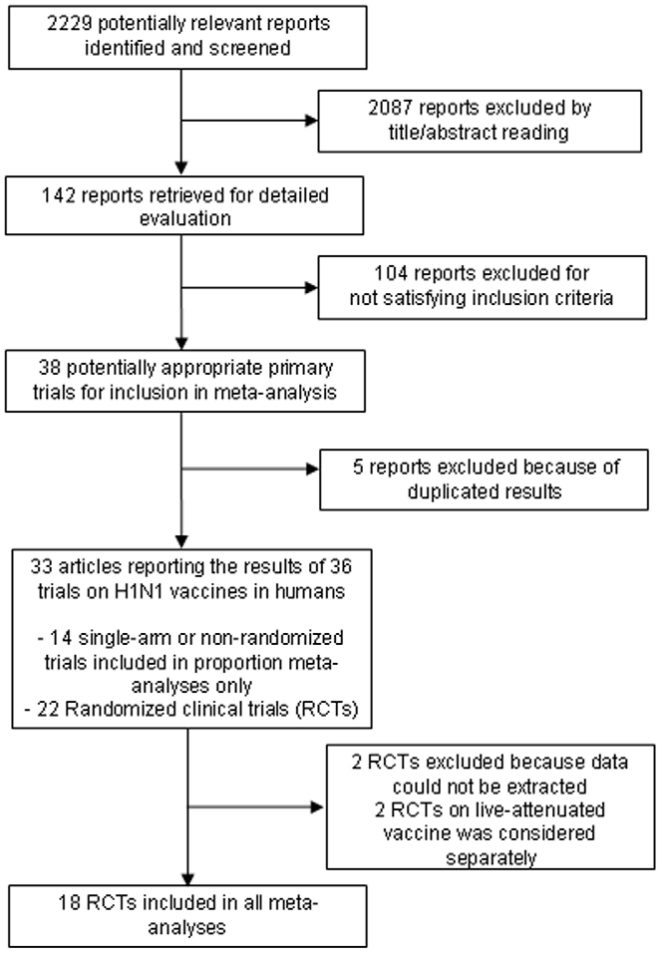
Flow diagram for the meta-analysis.

Of the 2229 papers initially retrieved (**Figure 1**), 33 reported the results of 36 studies evaluating the immunogenicity and/or safety of 2009 H1N1 vaccination in humans [8], [9], [11], [12], [13], [14], [15], [16], [17], [18], [19], [20], [21], [22], [23], [24], [25], [26], [27], [28], [29], [30], [31], [32], [33], [34], [35], [37], [39], [54], [55], [56], [57], [58], [59]. Fourteen studies were single-arm (with respect to pandemic vaccines) or non-randomized trials and were thus included in proportion meta-analyses only [9], [13], [14], [16], [21], [22], [27], [31], [32], [34], [55], [57], [58]. One of the above papers also reported data on a randomized clinical trial (RCT) [55], as did the remaining 19 papers, which reported the results of another 21 RCTs [8], [11], [12], [15], [17], [18], [19], [23], [24], [25], [26], [28], [29], [30], [33], [35], [37], [39], [54], [59], for a total of 22 RCTs. Although we were able to obtain some additional information from corresponding authors in two cases [19], [30], two RCTs were excluded because data could not be extracted [24], and data on some outcomes were missing in some studies (**Table S1**). Two RCTs reported data on live-attenuated vaccine, which was considered substantially different from other inactivated vaccines and discussed separately [23]. The remaining 18 RCTs were included in all primary analyses and included a total of 76 arms; of those 73 had data on the HI primary outcome after the first/single dose of vaccine, and 61 after two doses. All RCTs were started in 2009 and evaluated a vaccine including the strain recommended by WHO (derived from A/California/7/2009), and 15 were sponsored by vaccine manufacturers (**Table S1**).

With one exception [8], all trials reported seroconversion data, which were provided by the authors in one case [19]. Based on their reporting, 10 RCTs accomplished 3 out of 3 quality criteria proposed by Juni et al. [42], 4 trials accomplished 2 criteria, 6 only fulfilled the requirement for dropout/withdrawals reporting, finally 2 trials “scored” 0 (**Table S1**). As regards the single items, the procedures for the generation of allocation sequences were correctly reported (and made) in 12 trials (54%); the methods of allocation concealment were appropriate in 14 studies (64%); and 18 studies (82%) correctly reported the number, reasons for and statistical handling of dropouts and withdrawals. Importantly, 6 trials did not report any blinding procedure and only 4 RCTs were double-blinded.

**Figure 2:**
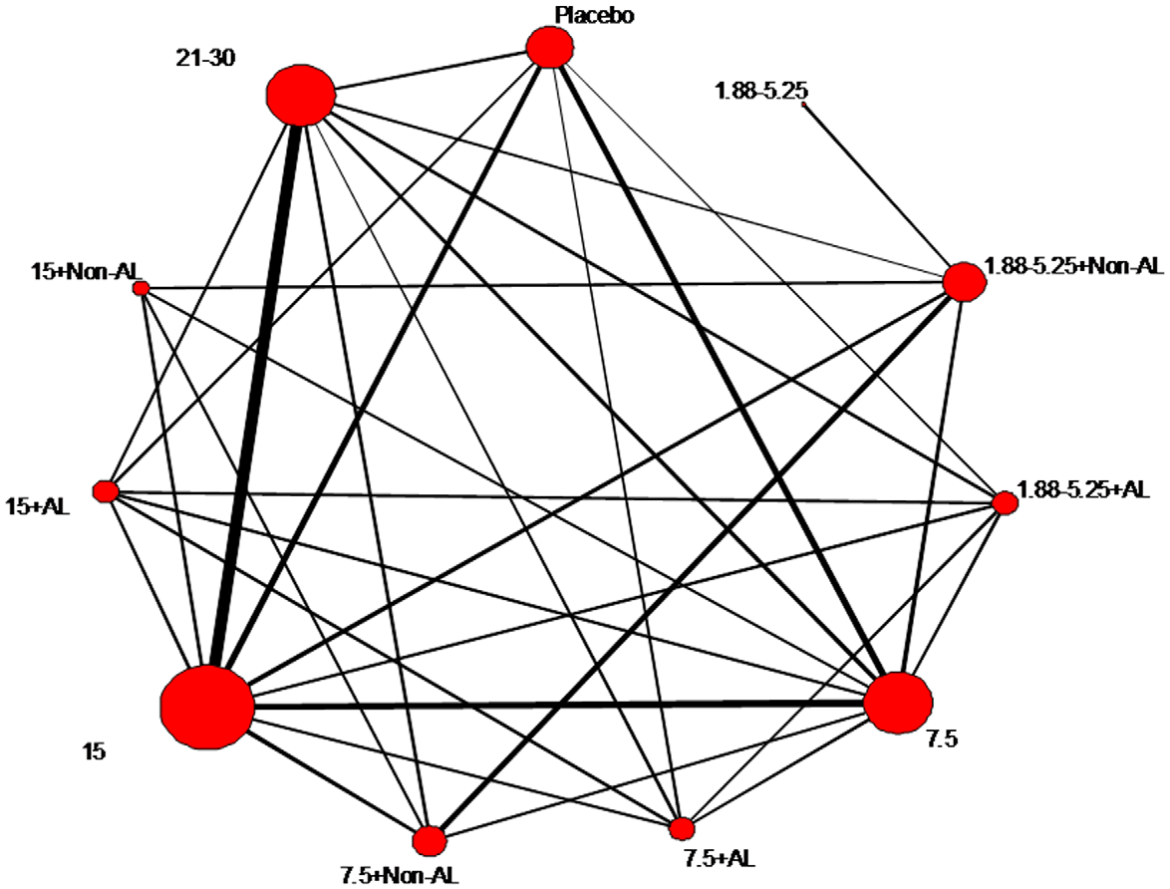
The network for multiple-treatments meta-analysis (MTM) includes all the randomized controlled trials that evaluate hemagglutinination-inhibition vaccine efficacy after the first or single dose. Each node represents a treatment and is named with letters depending on whether the vaccine included aluminum (AL), non-aluminium adjuvant (Non-AL), or no adjuvant; and with numbers reflecting the administered hemagglutinin antigen per dose (in µg): 1.88–5.25, 7.5, 15, or 21–30. The size of each node is proportional to the total number of patients randomized. Links represent head-to-head comparisons between the linked nodes with the thickness being proportional to the number of trials reporting on each specific comparison.

**Figure 2** shows the geometry of the trial network for HI after the first or a single dose of vaccine; the network after two doses being very similar. This is a network with high diversity where almost all formulations had been compared among themselves. The PIE indexes were 0.87 (one dose only) and 0.86 (two doses), suggesting large diversity in the networks, and the co-occurrence was formally statistically significant or close to significance, suggesting that there was strong preference for some comparisons (C-score p-values = 0.03 and 0.06 for single dose and two doses networks).

### Immunogenicity

Overall, the 18 RCTs included in primary analyses reported immunogenicity data on a total of 18,444 subjects, of whom 16,725 received at least one dose of vaccine.

When single arm results were examined (**Tables S2 and S3**), the immunogenicity tended to be higher in almost all trials among adults and adolescents than elderly and children. After two doses of split/subunit inactivated vaccine, seroconversion rates were > = 75% in all arms evaluating adults (n = 25) and adolescents (n = 12), whatever the dosage and adjuvant (if any). However, seroconversion rates were higher than 70% also in all arms evaluating elderly (n = 11) and children (n = 21), with one [11] and two [27] exceptions, respectively. Even after one dose only, among adults and adolescents almost all split/subunit formulations were able to confer a robust immunogenicity: seroconversion rate was higher or equal than 70% in all of the 41 (adults) and 13 (adolescents) trial arms, with only two [12], [57] and one [37] exceptions, respectively. Diversely, the seroconversion rate did not reach 70% in 9 arms out of 20 among the children, and 5 out of 20 among the elderly.

In direct-comparison meta-analyses of RCTs (**Table 1**), after a single dose of vaccine, both adjuvanted and non-adjuvanted low-dose formulations tended to show significantly lower immunogenicity than higher doses, with relative risks increasing on average about 1.05- and 1.10-fold, respectively with each dose step increase when all age groups were considered. The improvement with higher doses tended to be higher in children and in the elderly. Conversely, after two doses, there was hardly any dose-response that could be detected (relative risks on average were 1.01- and 1.05-fold per each dose step increase, respectively for adjuvanted and non-adjuvanted formulations).

**Table 1:** Direct meta-analyses comparing the immunogenicity (rates of seroconversion *) of H1N1 influenza vaccines at higher versus next lower doses of hemagglutinin antigen (HA).

	Adults 1		Elderly 2		Adolescents 3		Children 4		All ages
	RR (95%CI)	N (ref.)	RR (95%CI)	N (ref.)	RR (95%CI)	N (ref.)	RR (95%CI)	N (ref.)	RR (95%CI)
Non-adjuvanted vaccines									
15 vs 7.5 (1 dose only)	1.05 (1.02–1.09)	2334 [12], [19], [29], [33]	1.07 (1.00–1.14)	1583 [19], [29], [33]	1.00 (0.97–1.03)	1309 [19]	1.07 (1.00–1.15)	1755 [19], [28]	1.05 (1.01–1.09)
30 vs 15 (1 dose only)	1.02 (1.00–1.06)	3607 [11], [15], [18], [19], [29], [33]	1.12 (1.04–1.22)	2447 [11], [17], [19], [29], [33]	1.02 (1.00–1.03)	2094 [8], [19]	1.08 (1.05–1.12)	2481 [8], [19], [26], [54]	1.05 (1.03–1.08)
All dosages ** (1 dose only)	1.03 (1.01–1.06)	4320	1.09 (1.04–1.15)	2890	1.01 (1.00–1.03)	2312	1.08 (1.04–1.12)	3123	1.05 (1.03–1.07)
15 vs 7.5 (2 doses)	0.99 (0.97–1.01)	1481 [12], [19]	1.09 (1.02–1.16)	848 [19]	1.00 (0.99–1.02)	1112 [19]	1.03 (0.98–1.09)	1559 [19], [28]	1.02 (0.99–1.04)
30 vs 15 (2 doses)	1.02 (0.98–1.06)	2561 [11], [15], [18], [19]	1.14 (0.88–1.47)	1588 [11], [18], [19]	1.00 (1.00–1.01)	1763 [8], [19]	1.00 (0.99–1.01)	2174 [8], [19], [26], [54]	1.01 (1.00–1.02)
All dosages ** (2 doses)	1.00 (0.98–1.02)	2917	1.11 (0.98–1.25)	1725	1.00 (0.99–1.01)	1958	1.01 (0.99–1.04)	2762	1.01 (1.00–1.03)
Adjuvanted vaccines									
7.5 vs 1.88–5.25 (1 dose only)	0.96 (0.84–1.08)	593 [12], [19], [55]	–	0	1.28 (0.91–1.79)	58 [37] Sq	1.53 (1.01–2.33)	58 [37] Sq	1.02 (0.89–1.17)
	0.96 (0.83–1.11)	307 [19] Al							
	0.93 (0.72–1.19)	286 [12], [55] Sq							
15 vs 7.5 (1 dose only)	1.16 (1.06–1.28)	486 [12], [19]	1.31 (1.07–1.59)	216 [19] Al	1.12 (1.03–1.22)	407 [19] Al	1.29 (1.11–1.50)	398 [19] Al	1.18 (1.11–1.26)
	1.15 (1.03–1.28)	410 [19] Al							
	1.25 (0.99–1.57)	76 [12] Sq							
All dosages ** (1 dose only)	1.04 (0.96–1.12)	925	1.16 (0.92–1.48)	324	1.10 (1.02–1.17)	570	1.32 (1.14–1.52)	456	1.10 (1.04–1.17)
7.5 vs 1.88–5.25 (2 doses)	1.02 (0.92–1.12)	347 [12], [19]	–	0	1.00 (0.94–1.07)	58 [37] Sq	1.00 (0.94–1.07)	58 [37] Sq	1.00 (0.96–1.05)
	1.03 (0.91–1.17)	298 [19] Al							
	1.00 (0.84–1.18)	49 [12] Sq							
15 vs 7.5 (2 doses)	1.17 (1.08–1.26)	396 [19] Al	0.99 (0.88–1.10)	204 [19] Al	1.07 (1.00–1.14)	375 [19] Al	1.04 (1.00–1.09)	389 [19] Al	1.07 (1.01–1.13)
All dosages ** (2 doses)	1.08 (1.02–1.14)	639	1.06 (0.95–1.19)	308	1.04 (1.00–1.09)	523	1.03 (0.98–1.07)	447	1.05 (1.02–1.07)

Only randomized trials were included. All doses are in µg. N = total number of subject analyzed; (ref)  =  References to included studies; RR =  Random-effect risk ratio; CI  =  Confidence Intervals.

1 Adults  =  from 18 to 64 years;

2 Elderly  =  from 65 years;

3 Adolescents  =  from 10 to 17 years;

4 Children  =  from 6 months to 9 years (see Table S1 for several exceptions).

*Seroconversion  =  subjects with a pre-vaccination hemagglutinination-inhibition antibody titer < = 1∶10 and a post-vaccination titer > = 1∶40, or a pre-vaccination titer > = 1∶10 and an increase in the titer by a factor of four or more after vaccination.

**The total sample of overall meta-analyses may be lower (higher) than the sum of stratified meta-analyses because some arms had to be split to be included in more than a stratified meta-analysis (some arms were different from the main comparisons shown in the Table). In any case, no patient was included more than once in any of the meta-analyses. Al  =  Only aluminum adjuvants; Sq  =  Only squalene-based (oil-in-water) adjuvants.

We also tried to quantify the increase in immunogenicity provided by a second dose of vaccine computing the absolute percentages of subjects who achieved seroconversion only after the second dose for the most common formulations (**Table 2**). With the exception of low-dose adjuvanted formulations, no relevant improvements were associated with the administration of a second dose among the adults, adolescents and elderly (additional seroconversions ranging from 2% to 14%). By contrast, among children the second dose substantially increased seroconversion rates for any formulation (min. +19%; max +37%), with the exception of high-dose non-adjuvanted vaccines (+9%).

**Table 2:** Absolute percentages of subjects who did not achieve seroconversion * after the first dose of H1N1 influenza vaccination and who achieved seroconversion after the second dose, according to vaccine dose (in µg of Hemagglutinin antigen) and formulation (presence or absence of an adjuvant).

	Adults 1		Elderly 2		Adolescents 3		Children 4		All ages
Formulations**	% (95%CI)	N (ref)	% (95%CI)	N (ref)	% (95%CI)	N (ref)	% (95%CI)	N (ref)	% (95%CI)
**Non-adjuvanted**									
7.5×2 vs 7.5×1 All ***	8 (6–11)	465 [12], [19], [25]	8 (4–12)	170 [19], [25]	3 (1–6)	195 [19]	36 (24–49)	501 [19], [21], [27], [28]	22 (10–38)
15×2 vs 15×1 (All split-virus)	6 (2–9)	1504 [11], [12], [15], [18], [19], [39]	5 (0–14)	871 (10, 16–18)	4 (1–7)	1151 [8], [19], [21], [27], [54]	23 (15–31)	1556 [8], [19], [21], [26], [27], [28], [54]	11 (6–17)
30×2 vs 30×1 (All split-virus)	2 (0–6)	1147 [11], [15], [18], [19]	4 (0–8)	716 [11],	2 (0–3)	769 [8], [19], [54]	9 (4–17)	969 [8], [19], [26], [54]	3 (1–6)
**Adjuvanted**									
≤6×2 vs ≤6×1 All	5 (3–7)	597 [12], [16], [19], [25], [30], [31], [39]	18 (11–26)	104 [25], [31]	38 (22–56)	29 [37]	19 (4–49)	175 [9], [37]	7 (3–13)
- Aluminum	4 (1–10) $	98 [19]	–	0	–	0	–	0	4 (1–10)
- Other adjuvants	5 (2–8)	499	18 (11–26)	104	38 (22–56)	29	19 (4–49)	175	8 (3–15)
7.5×2 vs 7.5×1 All	14 (4–27)	224 [12], [19]	29 (20–39)	103 [19], [38]	8 (1–21)	266 [8], [19], [37], [54]	20 (3–46)	271 [8], [19], [37], [54]	13 (2–31)
- Aluminum	9 (6–14)	200 [19]	29 (20–39)	103	9 (5–14)	192 [19]	37 (30–44)	195 [19]	20 (17–23)
- Other adjuvants	21 (7–42)	24	–	0	7 (2–39)	74	12 (0–38)	76	11 (0–38)
15×2 vs 15×1 (All aluminum)	12 (8–18)	196 [19]	10 (5–18)	101 [19]	6 (3–9)	183 [19]	24 (19–31)	194 [19]	13 (11–16)

Data from single studies have been combined using proportion meta-analysis (random-effect model). Non randomized and randomized trials were included.

N  =  total number of subject analyzed; (ref)  =  References to included studies; CI  =  Confidence Intervals;

1 Adults  =  from 18 to 64 years;

2 Elderly  =  from 65 years;

3 Adolescents  =  from 10 to 17 years;

4 Children  =  from 6 months to 9 years (see Table S1 for several exceptions).

*Seroconversion  =  subjects with a pre-vaccination hemagglutinination-inhibition antibody titer < = 1∶10 and a post-vaccination titer > = 1∶40, or a pre-vaccination titer > = 1∶10 and an increase in the titer by a factor of four or more after vaccination.

**7.5×1  =  Results collected after the first or single dose of 7.5 µg; 7.5×2  =  Results collected after the second dose of 7.5 µg.

***All split-virus except 133 adults and 33 elderly subjects receiving whole-virus, with similar results [25].

$ Whole-virus only.

The potential impact of adjuvants was first explored in direct meta-analyses of RCTs comparing adjuvanted versus non-adjuvanted vaccines at any dosage, and separately at the most frequently used dosages (7.5 µg and 15 µg – **Table 3**). The use of aluminum did not seem to provide any benefit at any age, especially after one dose only: some aluminum-adjuvanted formulations were significantly less immunogenic than non-adjuvanted ones at equal dose. Very scarce data were available on adjuvants other than aluminum, because most of the RCTs testing vaccines with oil-in-water adjuvants only compared adjuvanted and non-adjuvanted preparations at different doses (being thus excluded from head-to-head comparisons of equal-dose vaccines).

**Table 3:** Direct meta-analyses comparing the immunogenicity (rates of seroconversion *) of adjuvanted versus non-adjuvanted H1N1 influenza vaccines.

Adjuvanted vs Non-adjuvanted, by age	Adults 1		Elderly 2		Adolescents 3		Children 4		All ages
	RR (95%CI)	N (ref.)	RR (95%CI)	N (ref.)	RR (95%CI)	N (ref.)	RR (95%CI)	N (ref.)	RR (95%CI)
7.5 (1 dose only)	0.84 (0.73–0.98)	607 [12], [19]	0.72 (0.60–0.86)	255 [19] Al	0.83 (0.77–0.89)	421 [19] Al	0.73 (0.63–0.84)	430 [19] Al	0.80 (0.74–0.86)
	0.81 (0.74–0.89)	531 [19] Al							
	0.98 (0.72–1.33)	76 [12] Sq							
15 (1 dose only)	1.18 (0.62–2.26)	1532 [12], [19]	0.89 (0.79–1.00)	950 [19] Al	0.93 (0.88–0.98)	1295 [19] Al	0.90 (0.82–0.98)	1313 [19] Al	0.92 (0.86–0.98)
	0.88 (0.83–0.94)	1482 [19] Al							
	1.69 (1.13–2.53)	50 [12] Sq							
All doses, by age ** (1 dose only)	1.00 (0.88–1.14)	3362 [12], [19], [39]	0.83 (0.75–0.92)	1992 [19] Al	0.91 (0.84–0.99)	2684 [19] Al	0.82 (0.67–1.00)	1743 [19] Al	0.90 (0.85–0.95)
	0.88 (0.81–0.95)	3109 [19] Al							
	1.28 (0.99–1.67)	253 [12], [39] Sq							
7.5 (2 doses)	0.97 (0.69–1.35)	556 [12], [19]	0.98 (0.89–1.08)	240 [19] Al	0.89 (0.85–0.94)	387 [19] Al	0.95 (0.91–0.99)	415 [19] Al	0.92 (0.86–0.99)
	0.83 (0.78–0.89)	509 [19] Al							
	1.17 (0.92–1.50)	49 [12] Sq							
15 (2 doses)	0.98 (0.94–1.02)	1321 [19] Al	0.89 (0.81–0.96)	812 [19] Al	0.95 (0.92–0.99)	1100 [19] Al	0.99 (0.96–1.01)	1165 [19] Al	0.96 (0.93–1.00)
All doses, by age ** (2 doses)	1.01 (0.91–1.12)	2957 [12], [19], [39]	0.95 (0.88–1.03)	1711 [19] Al	0.95 (0.89–1.02)	2294 [19] Al	0.97 (0.94–1.01)	1580 [19] Al	0.97 (0.93–1.00)
	0.95 (0.82–1.10)	2783 [19] Al							
	1.17 (1.06–1.28)	174 [12], [39] Sq							

Adjuvanted vs Non-Adjuvanted, by adjuvant type	Aluminum		MF-59		AS03_(A or B)_		All adjuvants
	RR (95%CI)	N (ref.)	RR (95%CI)	N (ref.)	RR (95%CI)	N (ref.)	RR (95%CI)
After one dose only – All ages, equal doses being compared **	0.87 (0.83–0.92)	9528 [19]	1.27 (0.74–2.17)	126 [12]	1.34 (1.13–1.60)	127 [39]	0.90 (0.85–0.95)
After two doses – All ages, equal doses being compared **	0.96 (0.92–0.99)	8368 [19]	1.17 (0.92–1.50)	47 [12]	1.17 (1.05–1.29)	127 [39]	0.97 (0.94–1.00)

Only randomized trials were included. All doses are in µg. N  =  total number of subject analyzed; (ref)  =  References to included studies; RR =  Random-effect risk ratio; CI  =  Confidence Intervals; Ad  =  Adjuvant.

1 Adults  =  from 18 to 64 years;

2 Elderly  =  from 65 years;

3 Adolescents  =  from 10 to 17 years;

4 Children  =  from 6 months to 9 years (see Table S1 for several exceptions).

*Seroconversion  =  subjects with a pre-vaccination hemagglutinination-inhibition antibody titer < = 1∶10 and a post-vaccination titer > = 1∶40, or a pre-vaccination titer > = 1∶10 and an increase in the titer by a factor of four or more after vaccination.

**The total sample of overall meta-analyses may be lower (higher) than the sum of stratified meta-analyses because some arms had to be split to be included in more than a stratified meta-analysis (some arms were different from the main comparisons shown in the Table); in any case, no subject was counted twice. Al  =  Only aluminum adjuvants; Sq  =  Only squalene-based (oil-in-water) adjuvants.

Both the potential impact of adjuvants and higher doses were further evaluated in MTM. **Table 4** shows the summary risk differences (RDs) and relative 95% credibility intervals (95%CI) for hemagglutinin-inhibition, using the 1.88–5.25 µg + other adjuvant (non aluminum) formulation as the baseline comparator. That baseline comparator formulation achieved immunogenicity in 83.4% and 96.5% of the vaccinated people, after a first/single and two doses, respectively. Overall, after the first or single dose, the formulation including oil-in-water adjuvants and 1.88–5.25 µg showed significantly higher immunogenicity than all non-adjuvanted and aluminum-adjuvanted preparations. As regards the other formulations that also included oil-in-water adjuvants, the use of higher doses of HA (7.5 µg and 15 µg) did not provide major improvement. The results after two doses were quite similar, and oil-in-water 1.88–5.25 µg formulations had the best immunogenicity estimate. Both networks consist of loops formed primarily from studies with multiple arms (up to seven), which by definition are consistent. Moreover, the model assuming consistency showed a better trade-off between model fit and complexity thus supporting the assumption of consistency.

**Table 4:** Results of the multiple-treatments meta-analysis comparing different influenza 2009 H1N1 formulations.

	Hemagglutinination-Inhibition	
Vaccine Formulations *	One (or single) dose	Two doses
	RD (95% CI)	RD (95% CI)
1.88–5.25 + non-aluminum adjuvants	Ref. Category	Ref. Category
Placebo	−0.82 (−0.83; −0.81)	−0.96 (−0.96; −0.94)
1.88–5.25	−0.41 (−0.67; −0.13)	−0.88 (−0.96; −0.26)
1.88–5.25 + aluminum	−0.41 (−0.60; −0.23)	−0.65 (−0.85; −0.39)
7.5	−0.24 (−0.36; −0.13)	−0.25 (−0.42; −0.13)
7.5 + aluminum	−0.50 (−0.64; −0.36)	−0.52 (−0.76; −0.27)
7.5 + non-aluminum adjuvants	−0.00 (−0.08; +0.07)	−0.10 (−0.27; 0.00)
15	−0.18 (−0.31; −0.08)	−0.16 (−0.31; −0.05)
15 + aluminum	−0.34 (−0.52; −0.19)	−0.34 (−0.60; −0.13)
15 + non-aluminum adjuvants	+0.06 (−0.10; +0.15)	No data available
21–30	−0.08 (−0.19; +0.01)	−0.11 (−0.02; −0.24)

Only randomized trials were included.

*Numbers refer to doses of Hemagglutinin antigen in micrograms. Risk differences (RD), with their respective 95% credible intervals (CI), compared with a vaccine including 1.88–5.25 µg hemagglutinin antigen and non-aluminum adjuvants (baseline comparator). RD expresses the absolute difference in risk between the two groups that is attributable to the intervention—i.e., if the likelihood of seroconversion in the reference group is 0.80 and it is 0.50 in the experimental group, the risk difference will be 0.80−0.50 = 0.30. The baseline value for hemagglutination–inhibition is 83.4% after the first or single dose; 96.5% after two doses. Aluminum adjuvants were aluminum hydroxide or aluminum phosphate. Non-aluminum adjuvants were oil-in-water emulsions.

The results of the proportion meta-analyses, although merely descriptive, are in line with those from direct-comparison meta-analyses and MTM, with seroconversion rates being higher for oil-in-water adjuvanted vaccines and after two doses in almost all formulations and at all ages (**Table S4**).

### Adverse events

The methodology adopted to record adverse events in each study, along with the definitions and reporting issues, have been reported in detail in **Table S5**. Data on any local and any systemic adverse events could not be extracted from several RCTs (13 out of 22 RCTs), whereas all studies provided details on serious adverse events (any medical occurrence that resulted in death, life-threatening medical conditions, persistent or substantial disability or incapacity, or admission to hospital). There were no deaths and three serious vaccine-related adverse events were reported in 34 randomized and non-randomized trials (**Table S1**): one adult with multiple allergies and mastocytosis had symptoms of immediate allergy consistent with anaphylaxis 1 hour after immunization with a non-adjuvanted vaccine containing 21 µg of HA [30]; one 8-year old child experienced a 4-day episode of fluctuating fever (to 39.7°C) with onset within 24 hours of the first 30-µg dose of a non-adjuvanted formulation [26]; finally one 11-month old child had a reactive knee arthritis in the leg in which a 1.875 µg AS03-adjuvanted vaccine had been administered two days previously [35]. All cases made a full recovery within ten days at most. Given that all trials included a total of 22,826 subjects receiving a 2009 H1N1 vaccine, the overall rate of reported vaccine-related serious adverse events was 0.013%.

As regards the most relevant mild or moderate solicited adverse events (local any, systemic any, fever and injection-site pain), we again used both proportion and direct-comparison meta-analyses (including all trials and randomized trials only, respectively), the results of which are reported for each vaccine formulation and after each dose in the online supplemental Tables. After both first and second vaccine dose, the absolute rates of the selected adverse events varied widely across formulations, but differences in the age-range of included studies, and outcome definition and severity thresholds are certainly also responsible for the wide ranges observed: fever ranged from 0% to 15%; local pain 7%–84%; systemic any 1%–84%; and local any 0%–57%.

In proportion meta-analyses, formulations including oil-in-water adjuvants generally showed the highest rates of pain and any local and systemic adverse events (**Table S6**). Such a difference was confirmed in direct-comparison meta-analyses for pain only, while no or very scarce data were available for fever, local and systemic adverse events (**Tables S7**, **S8**, **S9**, **and S10**). Direct-comparison meta-analyses also showed that, compared with the non-adjuvanted formulations, the aluminum adjuvanted formulations at the same dose significantly increased the risk of local harms and pain specifically.

In contrast to adjuvanted vaccines, non-adjuvanted formulations showed a moderated dose-response for all adverse events (**Table S6**), although such a trend was found to be significant only in some of the direct-comparison meta-analyses on fever and any systemic adverse events (**Tables S7**, **S8**, **S9**, **and S10**).

Proportion meta-analyses also showed that there was no strong evidence of an increase in the risk of any of the harms when two doses were administered rather than a single one, for all of the formulations considered (**Table S11**).

## Discussion

Our analysis summarizes what we have learnt from trials on the immunogenicity and harms of 2009 H1N1 vaccines. First, after two doses, all 2009 H1N1 split or subunit inactivated vaccines were highly immunogenic and overcome the seroconversion criteria set by both CPMP [45] and US FDA [60], with seroconversion rates below 70% in only three out of 69 evaluated trial arms. Second, after one dose only, all split or subunit inactivated vaccines were able to induce a satisfactory immunogenicity (> = 70%) in adults and adolescents, while only some formulations showed acceptable results for children and elderly (non-adjuvanted at high-doses and oil-in-water adjuvanted vaccines). Indeed, a second dose of vaccine did not seem to substantially increase the immunogenicity among adults and adolescents, but it may definitively be needed for children (especially if a low-dose non-adjuvanted vaccine is administered), and results remain controversial for the elderly. Third, vaccines with oil-in-water adjuvants were more immunogenic than both non-adjuvanted and aluminum-adjuvanted vaccines at equal doses (even at higher doses in many cases). In agreement with H5N1 Avian vaccines [43], 2009 H1N1 vaccines did not seem to obtain any benefit from the inclusion of aluminum. Fourth, higher doses were slightly more immunogenic in non-adjuvanted formulations, especially for the children and the elderly, for whom the lowest possible dose inducing a satisfactory immunogenicity after a single dose of vaccine was 15 µg. In contrast, for adjuvanted vaccines the seroresponse rate of doses lower than 6 µg (even with as little as 1.875 µg of HA) was not significantly lower than that achieved after higher doses.

Finally, in agreement with large observational surveillance studies [61], [62], the rate of serious vaccine-related adverse events was low for all 2009 H1N1 vaccines (0.013% overall). Theoretically, however, if we consider that over 1 billion subjects are to be vaccinated with 2009 H1N1 vaccines, such a rate would translate into 130,000 serious adverse events, which would need to be compared against the potential major benefits. On the other hand, we recognize that only 22,826 subjects were included and that the length of follow up was typically short, thus this meta-analysis does not have enough power to draw a conclusion for vaccine safety at the population level. Such an issue requires further investigation from future cost-benefit analyses based upon the emerging large observational studies. As regards mild to moderate adverse reaction, these were clearly more (and very) frequent for oil-in-water adjuvanted vaccines. It is worth noting, however, that the reporting of mild or moderate adverse events was lacking or suboptimal in many trials, and the results of such analyses must be interpreted with caution.

The practical implications of all the above findings depend on what type of vaccine is required in a particular moment. Clearly, during the 2009 pandemic, dose-sparing strategies were crucial, as vaccine provision had to be as large and quick as possible. In such a situation, vaccine including oil-in-water adjuvants may represent the best option, as they were able to induce the highest protection after a single, low dose of HA. If vaccination is considered a life-saving intervention, their higher rate of mild or moderate local reactions could be regarded as a clinically acceptable decrease in tolerability. In the current situation, however, in which 2009 H1N1 formulations have been added to trivalent seasonal vaccines [7], the need for the lowest possible dose may be lower, as opposed to tolerability. Therefore, also non-adjuvanted vaccines may represent a possible option, although a dose of over 20 µg would be preferable, even more for elderly and children.

Close to the end of this work, Yin et al. published a meta-analysis on the same topic [63]. It is difficult to compare the results of the two meta-analyses because of several important differences: Yin et al. ended the search in October 2010 and could not include 9 RCTs [11], [23], [25], [28], [33], [37], [39], [54], [55] and 9 non-randomized trials [13], [14], [16], [20], [22], [32], [55], [57], [58] that were included in the present analysis; only proportion meta-analyses were carried out, excepted some head-to-head comparisons on adjuvanted vs non-adjuvanted vaccines (which, however, almost totally depended on two large trials with some overlapped data [19], [36]); no quantitative analyses were made on adverse events; finally and most importantly, data from randomized and non-randomized trials at different hemagglutinin dosages were grouped together, thus no indications on the dose to use were given. Despite these differences, the authors reached similar conclusions regarding the effectiveness of different adjuvants.

So far, we have considered subunit or split inactivated vaccines, which compose almost all of the formulations that have been approved by both FDA and EMA and distributed during the past seasons [64], [65]. However, alternative formulations were also tested. Two RCTs evaluated the immunogenicity of an adjuvanted, whole-virus, live-attenuated vaccine [23]. The seroconversion rates, however, were not encouraging for both children and adults (<30% after two doses). Four RCTs compared whole-virus versus split-virion inactivated vaccines [19], [25], [35]. In a meta-analysis, whole-virus vaccines showed no differences in immunogenicity after the first dose, while after the second dose they showed significantly lower rates of seroconversion (summary RR 1.39; 95% CI: 1.11–1.73 – data not shown). The comparison, however, was problematic, because the doses of the whole- and split-virus vaccines being compared were different in all trials. Also, non-adjuvanted whole-virus preparations were compared to adjuvanted split-virus vaccines, and vice versa. Therefore, we cannot conclude on an inferior immunogenicity of whole-virus as compared to split-virus vaccines.

Some authors suggested that, at least for the children, previous (or simultaneous) vaccination with the seasonal trivalent influenza vaccine might decrease the antibody response to the 2009 H1N1 vaccines [26]. This issue was evaluated in five other trials, but we could not perform a meta-analysis because of large differences in vaccine formulation (and study design) [14], [20], [25], [34], [55]. However, none of these trials found evidence that receipt of the seasonal vaccine decreases the likelihood of achieving immunogenic protection after H1N1 vaccination. Given that four of the above trials included adults only [20], [25], [34], [55], the impact of previous seasonal vaccination on H1N1 vaccine response is likely to be unsubstantial for subjects over 18 years of age, but uncertainty remains for the children. Indeed, this is not a secondary issue and requires further consideration.

As previously mentioned, we could not formally test the influence of quality on summary estimates using head-to-head meta-analyses, because of the few RCTs that were classified at higher risk of bias, and the already high number of required stratifications of the analyses. However, we carried out proportion meta-analyses, which included also non-randomized trials, stratifying by quality (**Table S12**). Overall, it was not possible to note a clear trend, with studies at higher risk of bias showing sometimes higher sometimes lower summary estimates than trials at lower risk of bias (and most confidence intervals overlapping). However, we should caution that reported quality is only a surrogate of the true quality of a trial [66], and it is possible that some trials may thus have been misclassified in terms of their quality scores. In any case, also taking into account the pressure due to the pandemic, a higher quality of the reporting should be requested in the future.

Publication bias is a potential threat to the validity of all meta-analyses, especially when data are fragmented as in the present case, and no funnel plots or formal tests are meaningful [67], [68]. We made any effort to locate all published trials, and we searched 9 clinical trial registries to locate all launched RCTs from the pandemic start to April, 2010 (a year before the end of the search of published trials). We found that only 21 out of 68 (30.8%) registered RCTs on H1N1 vaccines had been published in peer-reviewed journals, representing 48.1% of the randomized sample size (19875 of 41329) [Ioannidis et al., submitted]. Therefore, the potential for publication bias does exist and should be taken into account when interpreting the results of the study.

Our study has some other potential limitations that must be mentioned. First, most of the published trials were conducted under sponsorship by the companies developing the vaccines. Thus it is very difficult to draw any firm conclusions about the potential impact of any sponsorship bias, even if the limited data showed no clear patterns (**Tables S2 and S3**). In any case, the conduct of large trials by non-industry agencies would help curtail the possibility that sponsorship bias may result in inflated estimates of immunogenicity. Second, all direct comparisons between adjuvanted and non-adjuvanted vaccines were based upon three RCTs only, and in particular the conclusion of a superior immunogenicity of non-aluminum as compared to aluminum formulations was based only upon indirect comparisons, as no published study has directly compared two different types of adjuvants. In any case, searching in clinical trial registries, we found only one Brazilian RCT that compared various adjuvants (NCT01111968). This study was completed in March 2011 and the results may be published in the near future. Third, no study compared oil-in-water adjuvants by different companies, and further randomized trials are thus needed. Finally, the degree to which immunological measures can be considered markers of vaccines effectiveness is not precisely known, and whether the observed differences in immunogenicity ultimately led to better protection for H1N1 vaccines is unclear [69]. In particular, some authors suggested that HI titers could not be well correlated with protection against influenza-like illnesses for live-attenuated vaccines, as vaccine efficacy was reported in the absence of high seroprotection rates [70], [71]. Therefore, although HI responses were modest compared to levels achieved by inactivated vaccines [23], we cannot discard the use of live-attenuated vaccines, and further studies are needed on the topic.

## Supporting Information

Table S1 **Characteristics of published studies investigating the immunogenicity and/or safety of 2009 H1N1 influenza vaccination.**
(PDF)

Table S2 **Rates of seroconversion (according to hemagglutinination-inhibition) after the first or single dose of 2009 H1N1 vaccine in each of the retrieved studies.**
(PDF)

Table S3 **Rates of seroconversion (according to hemagglutinination-inhibition) after two doses of 2009 H1N1 vaccine in each of the retrieved studies.**
(PDF)

Table S4 **Rates of seroconversion after H1N1 influenza vaccination, according to vaccine dose (in µg of hemagglutinin antigen) and formulation (presence or absence of an adjuvant; one administration or two).** Data from single studies have been combined using proportion meta-analysis (random-effect model). Non randomized and randomized trials were included.
(PDF)

Table S5 **Adverse events: Methods of assessment, definitions and reporting issues of each included trial.**
(PDF)

Table S6 **Rates of injection-site pain, fever, any systemic and local adverse event after one or two doses of 2009 H1N1 influenza vaccination, according to vaccine dose and formulation (presence or absence of an adjuvant).** Data from single studies have been combined using proportion meta-analysis (random-effect model).
(PDF)

Table S7 **Direct meta-analyses comparing the rates of pain at the injection site of H1N1 influenza vaccines at higher versus next lower doses of hemagglutinin antigen (HA) and of adjuvanted versus non-adjuvanted H1N1 influenza vaccines.** All doses are reported as µg of HA.
(PDF)

Table S8 **Direct meta-analyses comparing the rates of fever of H1N1 influenza vaccines at higher versus next lower doses of hemagglutinin antigen (HA) and of adjuvanted versus non-adjuvanted H1N1 influenza vaccines.** All doses are reported as µg of HA.
(PDF)

Table S9 **Direct meta-analyses comparing the rates of any systemic adverse event of H1N1 influenza vaccines at higher versus next lower doses of hemagglutinin antigen (HA) and of adjuvanted versus non-adjuvanted H1N1 influenza vaccines.** All doses are reported as µg of HA.
(PDF)

Table S10 **Direct meta-analyses comparing the rates of any local adverse event of H1N1 influenza vaccines at higher versus next lower doses of hemagglutinin antigen (HA) and of adjuvanted versus non-adjuvanted H1N1 influenza vaccines.** All doses are reported as µg of HA.
(PDF)

Table S11 **Absolute percentages of subjects who reported one adverse event (injection-site pain, fever, any systemic or any local) after the second dose of 2009 H1N1 vaccine, and who did not report such event after the first dose (according to vaccine dose and formulation – presence or absence of an adjuvant).** Data from single studies have been combined using proportion meta-analysis (random-effect model). All doses are reported as µg of hemagglutinin antigen.
(PDF)

Table S12 **Rates of seroconversion after H1N1 influenza vaccination, according to vaccine dose (in µg of hemagglutinin antigen), formulation (presence or absence of an adjuvant; one administration or two), and methodological quality (lower or higher risk of bias, see text for details).** Data from single studies have been combined using proportion meta-analysis (random-effect model). Non randomized and randomized trials were included.
(PDF)
