# Supplementary material for: Meta-Analysis of the Immunogenicity and Tolerability of Pandemic Influenza A 2009 (H1N1) Vaccines
Source: PLoS One. 2011 Sep 6;6(9):e24384. doi: 10.1371/journal.pone.0024384 (PMC3167852; doi:10.1371/journal.pone.0024384)
Supplement: Table S1 — Characteristics of published studies investigating the immunogenicity and/or safety of 2009 H1N1 influenza vaccination. (PDF) [file pone.0024384.s001.pdf]

Table S1. Characteristics of published studies investigating the immunogenicity and/or safety of 2009 H1N1 influenza vaccination.

First Author	Study Year	Country	Design	Age, years	Vaccine type	Dose given (n) in each arm*	Main outcomes extracted **	Quality ***	Sponsor
**RCTs**									
Arguedas [8,54,56]	2009	Costa Rica	RCT, No blinding	3-17	Inactivated, Surface-antigen	7.5+OA (54); 15 (83); 30 (57)	HI; Fever; Pain; Systemic any; Local any.	0, 0, 0	Novartis
Cheong [11]	2009	Korea	RCT Single blind	>=19	Inactivated, Split virus	15 (226); 30 (228)	HI; Fever; Pain.	0, 0, 1	Non-profit Institution
Clarck [12]	2009	UK	RCT, No blinding	18-50	Inactivated, Surface-antigen	3.75+OA (25); 7.5 (25); 7.5+OA (51); 15 (25); 15+OA (25)	HI; Fever; Pain.	0, 0, 1	Novartis
Gasparini 1 [55]	2009	Italy	RCT, No blinding	>=18	Inactivated, Surface-antigen	3.75+OA (69); 7.5+OA (141)	HI; Fever; Pain.	0, 0, 0	Novartis
Greenberg [15,59]	2009	Australia	RCT, Single blind	18-64	Inactivated, Split virus	15 (117); 30 (115)	HI; Fever; Pain; Systemic any; Local any.	1, 1, 1	CSL
Kung / Kao [17,18]	2009	Taiwan	RCT, Single blind	20-86	Inactivated, Split virus	15 (173); 30 (115)	HI; Fever; Pain; Systemic any; Local any.	1, 1, 1	Adimmune
Liang / Zhu / Wu [19,36,38]	2009	China	2 RCTs, Double blind	3-87	Inactivated, Split virus σ	Placebo (1432); 5+Al (99) σ; 7.5 (920); 7.5+Al (717); 10+Al (202) σ; 15 (4326); 15+Al (714); 30 (3419); 30+Al (318)	HI; Systemic any; Local any.	1, 1, 0	Hualan BB, Sinovac
Mallory [23]	2009	USA	2 RCTs, Double blind	2-49	Live-attenuated Whole virus	Placebo (128); 1.875+OA (514);	HI β; Systemic any; Fever.	1, 1, 1	MedImmune
Mironov 1 [24]	2009	Russia	RCT No blinding	18-60	Inactivated, Split virus	Placebo (10); Not reported (20)	Systemic any, local any	0, 0, 1	NPO Microgen
Mironov 2 [24]	2009	Russia	RCT No blinding	18-60	Intranasal, Whole virus	Placebo (10); Not reported (20)	Systemic any, local any	0, 0, 1	NPO Microgen
Nicholson [25]	2009	UK	RCT, Single blind	>=18	Inactivated, Whole vs split virus	3.75+OA (170); 7.5 (171) σ	HI; Fever; Pain.	1, 1, 1	Non-profit Institution
Nolan [26,59]	2009	Australia	RCT, Single blind	0.5-9	Inactivated, Split virus	15 (174); 30 (172)	HI; Fever; Pain; Systemic any; Local any.	1, 1, 1	CSL
Plennevaux [28,29]	2009	USA	2 RCTs Single-blind	>=0.5	Inactivated, Split virus	Placebo (145); 7.5 (445); 15 (445); 30 (242)	HI; Fever; Pain; Systemic any; Local any.	1, 1, 1	Sanofi
Roman 1 [30]	2009	Germany	RCT Single blind	18-60	Inactivated, Split virus	5.25+OA (56); 21 (61)	HI; Fever; Pain.	1, 1, 1	GSK
Roman 3 [39]	2009	Belgium	2 RCTs Single blind	18-60	Inactivated, Split virus	3.75 (63); 3.75+OA (124); 15 (66)	HI; Pain.	0,1,1	GSK
Talaat [33]	2009	USA	RCT Single blind	18-93	Inactivated, Split virus	Placebo (97); 7.5 (401); 15 (394); 30 (400)	HI; Fever; Pain; Systemic any; Local any.	0, 0, 1	CSL
Waddington [35]	2009	UK	RCT No blinding	0.5-12	Inactivated, Whole vs split virus	1.875+OA (392); 7.5 (414) σ	HI; Fever; Pain; Local any.	1, 1, 1	Non-profit Institution

Yasuda [37]	2009	Japan	RCT No blinding	0.5-19	Inactivated, Surface-antigen	3.75+OA (57); 7.5+OA (59)	HI; Fever; Pain.	0, 0, 1	Novartis
**Single-arm φ or non-randomized trials**									
Carmona [9]	2009	Spain	Single arm φ	0.5-3	Inactivated, Split virus	1.875+OA (101); 3.75+OA (50)	HI; Fever; Pain; Systemic any; Local any.	--, --, 0	GSK
Di [13]	2009	China	Single arm	19-55	Inactivated, Split virus	7.5 (95)	HI.	--, --, 0	Not reported
Esposito [14]	2009	Italy	Single arm φ	9-20	Inactivated, Surface-antigen	7.5+OA (36)	HI; Fever; Pain; Systemic any; Local any.	--, --, 1	Non-profit Institution
Gasparini 2 [55]	2009	Italy	Single arm	>=18	Inactivated, Surface-antigen	7.5+OA (154)	HI; Fever; Pain.	--, --, 0	Novartis
Igari [57]	2009	Japan	Single arm	20-64	Inactivated, Split virus	15 (389)	HI; Fever; Pain; Systemic any; Local any.	--, --, 1	Non-profit Institution
Ikematsu [16]	2009	Japan	Single arm	20-64	Inactivated, Split virus	3.75+OA (100); 15 (69)	HI; Fever; Pain.	--, --, 1	GSK
Loebermann [20]	2010	Germany	Single arm	>=18	Inactivated, Split virus	15 (69)	HI; Pain.	--, --, 1	Novartis
Lu [21]	2009	Taiwan	No randomiz. No blinding	1-17	Inactivated, Split virus	7.5 (58); 15 (122)	HI; Fever; Pain; Systemic any; Local any.	--, --, 1	Adimmune
Oh [27]	2009	Korea	No randomiz. No blinding	0.5-17	Inactivated, Split virus	7.5 (34); 15 (212)	HI; Fever; Pain.	-- ,--, 1	Non-profit Institution
Roman 2 [31]	2009	Belgium	Single arm φ	18-85	Inactivated, Split virus	3.75+OA (237)	HI; Fever; Pain.	--, --, 0	GSK
Scheifele [58]	2009	Canada	Single arm	0.5-3	Inactivated, Split virus	1.875+OA (152)	HI; Fever; Pain; Systemic any; Local any.	--, --, 0	Non-profit Institution
Sun [32]	2009	China	Single arm	18-60	Inactivated, Split virus	15 (58)	HI.	--, --, 1	Hualan BB
Vajo [34]	2009	Hungary	Single arm φ	>=18	Inactivated, Whole virus	5+Al (352)	HI; Fever; Pain; Systemic any; Local any.	--. --, 1	Omninvest
Madhun [22]	2010	Norway	Single arm	21-67	Inactivated, Split virus	3.75+OA (207)	HI; Fever; Pain; Systemic any; Local any.	--, --, 1	Non-profit Institution

* The dose shown refers to the amount of hemagglutinin antigen in micrograms; when two doses were administered at baseline, the total dose was doubled; Al = Aluminum adjuvant (Aluminum hydroxide or Aluminum phosphate); OA = Other adjuvant, either MF-59 [8,12,14,37,54,55], or AS03 [9,16,22,25,30,31,35,58]; In brackets the total n. of individuals in which immunogenicity (or safety for studies not assessing immunogenicity) was fully evaluated after the first dose (or the second if the results after the first dose were not reported). ** HI = Immunogenicity assessed using hemagglutinination-inhibition technique. *** Based upon the methodology described by Juni and coll. [42], the first number pertains to random generation of allocation sequences (0=non adequate; 1=adequate); the second to allocation concealment; the third to withdrawals/dropouts rate/description. φ These trials randomized participants into different groups, but for the aims of our analysis they were to be considered as single group trials (i.e. patients receiving two different doses both below 6 μg of HA; or patients randomized to receive either seasonal vaccine + pandemic vaccine, or the same pandemic vaccine only). σ Whole-virus vaccine. β This outcome was evaluated only in a subset of subjects.
