# Supplementary material for: Meta-Analysis of the Immunogenicity and Tolerability of Pandemic Influenza A 2009 (H1N1) Vaccines
Source: PLoS One. 2011 Sep 6;6(9):e24384. doi: 10.1371/journal.pone.0024384 (PMC3167852; doi:10.1371/journal.pone.0024384)
Supplement: Table S2 — Rates of seroconversion (according to hemagglutinination-inhibition) after the first or single dose of 2009 H1N1 vaccine in each of the retrieved studies. (PDF) [file pone.0024384.s002.pdf]

Table S2. Rates of seroconversion (according to hemagglutinination-inhibition) after the first or single dose of 2009 H1N1 vaccine in each of the retrieved studies.

Study (first author, reference)	1.88-5.25 % (n)	1.88-5.25 + Oil-in-water % (n)	1.88-5.25 + Aluminum % (n)	7.5 % (n)	7.5 + Aluminum % (n)	7.5 + Oil-in-water % (n)	15 % (n)	15 + Aluminum % (n)	15 + Oil-in-water % (n)	21-30 % (n)
**ADULTS - RCTs**										
Greenberg [15]							74 (120)			82 (119)
Clarck [12]		88 (25)		72 (25)		71 (51)	52 (25)		88 (25)	
Roman 1 [30]		98 (56)								95 (61)
Plennevaux [29]				92 (140)			96 (145)			97 (144)
Liang [19]			75 (99)	88 (323)	72 (208)		93 (137)	82 (202)		93 (991)
Kung [18]							92 (120)			92 (62)
Talaat [33]				82 (200)			88 (196)			95 (200)
Cheong [11]							89 (115)			95 (115)
Nicholson [25]		81 (133)		48 (137)*						
Gasparini [55]		91 (69)				94 (141)				
Roman 3A [39]		98 (60)					85 (66)			
Roman 3B [39]	70 (63)	94 (64)								
**ADULTS - Trials**										
Roman 2 [39]		95 (118)								
Vajo [34]			76 (200)							
Di Adults [13]				82 (95)						
Ikematsu [16]		94 (100)								
Loebermann [20]							84 (69)			
Sun [32]							86 (58)			
Madhun [22]		88 (207)								
Igari [57]							35 (389)			
Gasparini [55]						94 (50)				
**ELDERLY - RCTs**										
Plennevaux [29]				83 (95)			89 (100)			92 (98)
Liang [19]				80 (147)	57 (108)		84 (842)	75 (108)		91 (679)
Kao [17]							72 (53)			81 (53)
Talaat [33]				51 (201)			59 (198)			73 (200)
Cheong [11]							57 (111)			75 (113)
Nicholson [25]		51 (37)		29 (34)*						
**ELDERLY - Trials**										

Roman 2 [39]	79 (119)								
Vajo [34]		70 (152)							
Loebermann [20]						75 (63)			
Gasparini [55]					73 (154)				
**ADOLESCENTS - RCTs**									
Arguedas [8,54,56]					87 (45)	88 (77)			94 (52)
Liang [19]			96 (218)	80 (203)		96 (1091)	89 (204)		98 (863)
Yasuda [37]	62 (29)				79 (29)				
**ADOLESCENTS - Trials**									
Lu [21]						84 (31)			
Esposito [14]					100 (36)				
Oh [27]						78 (129)			
**CHILDREN - RCTs**									
Arguedas [8,54,56]					91 (46)	69 (75)			75 (48)
Plennevaux [28]			56 (210)			63 (200)			
Liang [19]			77 (232)	56 (198)		81 (1113)	73 (200)		88 (886)
Nolan [26,59]						87 (174)			94 (172)
Yasuda [37]	50 (28)				77 (30)				
**CHILDREN - Trials**									
Lu [21]			28 (58)			45 (91)			
Oh [27]			15 (34)			33 (83)			
Carmona [9]	99 (149)								
Scheifele [58]	84 (152)								

* Whole-virion vaccine. Studies reported in red have been sponsored by not-for-profit institutions. All other studies have been sponsored by vaccine manufacturers.
