# Supplementary material for: Meta-Analysis of the Immunogenicity and Tolerability of Pandemic Influenza A 2009 (H1N1) Vaccines
Source: PLoS One. 2011 Sep 6;6(9):e24384. doi: 10.1371/journal.pone.0024384 (PMC3167852; doi:10.1371/journal.pone.0024384)
Supplement: Table S3 — Rates of seroconversion (according to hemagglutinination-inhibition) after two doses of 2009 H1N1 vaccine in each of the retrieved studies. (PDF) [file pone.0024384.s003.pdf]

Table S3. Rates of seroconversion (according to hemagglutinination-inhibition) after <u>two doses</u> of 2009 H1N1 vaccine in each of the retrieved studies.

Study (first author, reference)	1.88-5.25 % (n)	1.88-5.25 + Oil-in-water % (n)	1.88-5.25 + Aluminum % (n)	7.5 % (n)	7.5 + Aluminum % (n)	7.5 + Oil-in-water % (n)	15 % (n)	15 + Aluminum % (n)	15 + Oil-in-water % (n)	21-30 % (n)
**<u>ADULTS - RCTs</u>**										
Greenberg [15]							82 (117)			90 (115)
Clarck [12]		92 (25)		78 (23)		92 (24)	75 (24)			
Roman 1 [30]		100 (56)								98 (61)
Liang [19]			79 (98)	97 (309)	81 (200)		96 (1125)	94 (196)		97 (857)
Kung [18]							93 (59)			92 (60)
Cheong [11]							89 (113)			94 (115)
Nicholson [25]		91 (129)		53 (133)*						
Roman 3A [39]		98 (59)					92 (66)			
Roman 3B [39]	86 (63)	100 (64)								
**<u>ADULTS - Trials</u>**										
Roman 2 [39]		98 (66)								
Ikematsu [16]		100 (100)								
Mallory [23]**		15 (222)**								
**<u>ELDERLY - RCTs</u>**										
Liang [19]				88 (137)	86 (103)		96 (711)	85 (101)		97 (144)
Kung [18]							71 (52)			80 (51)
Cheong [11]							59 (109)			78 (111)
Nicholson [25]		73 (37)		33 (33)*						
**<u>ELDERLY - Trials</u>**										
Roman 2 [39]		94 (67)								
**<u>ADOLESCENTS - RCTs</u>**										
Arguedas [8,54,56]						84 (45)	91 (77)			92 (52)
Liang [19]				99 (195)	89 (192)		99 (917)	95 (183)		99 (717)
Yasuda [37]		100 (29)				100 (29)				
**<u>ADOLESCENTS - Trials</u>**										
Lu [21]							87 (31)			

Oh [27]					86 (126)		
**CHILDREN - RCTs**							
Arguedas [8,54,56]				96 (46)	84 (75)		92 (48)
Plennevaux (28)		93 (190)			99 (178)		
Liang [19]		98 (220)	93 (195)		98 (178)	97 (194)	99 (756)
Nolan [26,59]					98 (159)		98 (165)
Waddington (35)	99 (392)	77 (414)*					
Yasuda [37]	100 (28)			100 (30)			
**CHILDREN - Trials**							
Lu [21]		75 (57)			80 (91)		
Oh [27]		56 (34)			62 (82)		
Carmona [9]	100 (147)						
Scheifele [58]	99 (146)						
Mallory [23]**	28 (143)**						

* Whole-virion vaccine. ** Live-attenuated vaccine. Studies reported in red have been sponsored by not-for-profit institutions. All other studies have been sponsored by vaccine manufacturers.
