# Supplementary material for: Meta-Analysis of the Immunogenicity and Tolerability of Pandemic Influenza A 2009 (H1N1) Vaccines
Source: PLoS One. 2011 Sep 6;6(9):e24384. doi: 10.1371/journal.pone.0024384 (PMC3167852; doi:10.1371/journal.pone.0024384)
Supplement: Table S4 — Rates of seroconversion after H1N1 influenza vaccination, according to vaccine dose (in µg of hemagglutinin antigen) and formulation (presence or absence of an adjuvant; one administration or two). Data from single studies have been combined using proportion meta-analysis (random-effect model). Non randomized and randomized trials were included. (PDF) [file pone.0024384.s004.pdf]

Table S4. Rates of seroconversion * after H1N1 influenza vaccination, according to vaccine dose (in μg of hemagglutinin antigen) and formulation (presence or absence of an adjuvant; one administration or two). Data from single studies have been combined using proportion meta-analysis (random-effect model). Non randomized and randomized trials were included.

	Adults [1]		Elderly [2]		Adolescents [3]		Children [4]		All ages
Formulations**	% (95%CI)	N (ref)	% (95%CI)	N (ref)	% (95%CI)	N (ref)	% (95%CI)	N (ref)	% (95%CI)
**Non-adjuvanted**									
7.5x1 – All	79 (65-90)	920 [12,13,19,25,29,33]	63 (41-82)	477 [19,25,29,33]	96 (93-98)	218 [19]	44 (21-69)	534 [19,21,27,29]	60 (45-73)
- Split-virus	85 (80-90)	783	72 (50-90)	443	96 (93-98)	218	44 (21-69)	534	62 (47-75)
- Whole-virus	48 (40-57)	137 [25]	29 (15-47)	34 [25]	--	0	--	0	44 (37-52)
7.5x2 – All	79 (38-100)	465 [12,19,25]	64 (11-100)	170 [19,25]	99 (96-100)	195 [19]	83 (68-94)	915 [19,21,27,28,35]	77 (60-91)
- Split-virus	90 (64-100)	332	88 (82-93)	137	99 (96-100)	195	84 (67-96)	501	83 (69-93)
- Whole-virus	53 (45-62)	133 [25]	33 (18-52)	33 [25]	--	0	77 (72-81)	414 [35]	64 (36-87)
15x1 (all split-virus)	81 (67-93)	2583 [11,12,15,18,19,20,29,32,33,39,57]	73 (61-84)	1367 [11,17,19,20,29,33,38]	90 (78-97)	1334 [8,19,21,27]	64 (47-79)	1754 [8,19,21,26,27,29]	74 (65-83)
15x2 (all split-virus)	89 (82-95)	1504 [11,12,15,18,19,39]	78 (44-98)	872 [11,18,19,38]	92 (79-99)	1151 [8,19,21,27]	90 (79-97)	1556 [8,19,21,26,27,29,54]	88 (80-94)
21-30x1 (all split-virus)	93 (90-95)	1692 [11,15,18,19,29,30,33]	83 (73-91)	1143 [11,17,19,29,33]	98 (96-100)	920 [8,19]	87 (79-94)	1111 [8,19,26]	89 (86-92)
21-30x2 (all split-virus)	94 (91-97)	1147 [11,15,18,19,30]	87 (67-98)	716 [11,18,19,38]	97 (86-100)	769 [8,19]	97 (94-99)	969 [8,19,26,54]	93 (88-97)
**Adjuvanted**									
1.88-5.25x1 – All	89 (84-94)	1131 [12,16,19,22,25,30,31,34,39,55]	69 (56-80)	308 [25,31,34]	62 (42-79)	29 [37]	82 (55-98)	329 [9,37,58]	87 (81-92)
- Aluminum	76 (71-80) [$]	299 [19,34]	70 (62-78) [$]	152 [34]	--	0	--	0	74 (70-78)
- Other adjuvants	92 (88-95)	832	66 (38-89)	156	62 (42-79)	29	82 (55-98)	329	89 (83-94)
1.88-5.25x2 – All	96 (90-99)	597 [12,16,19,25,30,31,39]	85 (60-99)	104 [25,31]	100 (88-100)	29 [37]	99 (98-100)	713 [9,35,37,58]	97 (94-99)
- Aluminum	79 (69-86)	98 [19]	--	0	--	0	--	0	79 (69-86)
- Other adjuvants	98 (94-100)	499	85 (60-99)	104	100 (88-100)	29	99 (98-100)	713	98 (96-100)
7.5x1 – All	80 (60-94)	450 [12,19,55]	65 (50-79)	262 [19,55]	91 (76-99)	320	76 (48-95)	292 [8,19,37]	83 (71-93)

						[8,14,19,37]			
- Aluminum [19]	72 (65-78)	208	57 (48-67)	108	80 (74-85)	203	56 (49-63)	198	68 (64-71)
- Other adjuvants	84 (56-99)	242	73 (65-80)	154	94 (80-100)	117	85 (67-97)	84	86 (74-95)
7.5x2 – All	83 (74-91)	224 [12,19]	86 (79-92)	103 [19,38]	91 (81-98)	266 [8,19,37]	95 (90-98)	271[8,19,37,54]	93 (84-98)
- Aluminum [19]	81 (75-86)	200	86 (79-92)	103	89 (83-93)	192	93 (88-96)	195	87 (85-90)
- Other adjuvants	92 (73-99)	24	--	0	93 (70-100)	74	97 (91-100)	76	95 (84-100)
15x1 – All	83 (77-87)	227 [12,19]	75 (66-83)	108 [19,38]	89 (84-93)	204 [19]	73 (66-97)	200 [19]	81 (78-83)
- Aluminum	82 (76-87)	202 [19]	75 (66-83)	108	89 (84-93)	204	73 (66-97)	200	80 (77-83)
- Other adjuvants	88 (69-97)	25	--	0	--	0	--	0	88 (69-97)
15x2 All Aluminum	94 (90-97)	196 [19]	85 (77-91)	101 [19,38]	94 (90-97)	183 [19]	97 (94-99)	194 [19]	94 (92-95)

N = total number of subject analyzed; (ref) = References to included studies; CI = Confidence Intervals; [1] Adults = from 18 to 64 years; [2] Elderly = from 65 years; [3] Adolescents = from 10 to 17 years; [4] Children = from 6 months to 9 years (see Table S1 for several exceptions). * Seroconversion = subjects with a pre-vaccination hemagglutinination-inhibition antibody titer <=1:10 and a post-vaccination titer >=1:40, or a pre-vaccination titer >=1:10 and an increase in the titer by a factor of four or more after vaccination. ** 7.5x1 = Results collected after the first or single dose of 7.5µg; 7.5x2 = Results collected after the second dose of 7.5µg. [$] Whole-virus only.
