# Supplementary material for: Meta-Analysis of the Immunogenicity and Tolerability of Pandemic Influenza A 2009 (H1N1) Vaccines
Source: PLoS One. 2011 Sep 6;6(9):e24384. doi: 10.1371/journal.pone.0024384 (PMC3167852; doi:10.1371/journal.pone.0024384)
Supplement: Table S5 — Adverse events: Methods of assessment, definitions and reporting issues of each included trial. (PDF) [file pone.0024384.s005.pdf]

Table S5. Adverse events: Methods of assessment, definitions and reporting issues of each included trial.

Trials	Fever	Pain at injection site	Any local adverse event	Any systemic adverse event	Method of assessment	Reporting issues
Arguedas [8,54]	Axillary temp. >38 °C	Mild: did not interfere with activity<br>Moderate: caused some impairment<br>Severe: affected daily activity and required medical attention	Any local reaction among: erythema, induration, swelling, pain	Any systemic reaction among: headache, fatigue, myalgia, arthralgia, chills, nausea, vomiting, diarrhea, fever	7-day self-compiled diary card	None
Carmona [9]	Axillary temp. ≥37.5 °C	Grade 1: minor reaction on touching site<br>Grade 2: cries/protests on touching site<br>Grade 3: cries when limb moved/spontaneously painful	Any local reaction among: redness, swelling, pain	Any systemic reaction among: drowsiness, irritability, loss of appetite, fever	7-day diary card compiled by parents	None
Cheong [11]	NR	Grade 1: does not interfere with activity<br>Grade 2: repeated use of non-narcotic pain reliever >24 h or interferes with activity<br>Grade 3: any use of narcotic pain reliever or prevents daily activity<br>Grade 4: emergency room visit or hospitalization	Any local reaction among: redness, swelling, pain, tenderness	Any systemic reaction among: fever, headache, malaise, shivering, fatigue, sweating, myalgia, and arthralgia	7-day self-compiled diary card	The dose to which the adverse events results were referred is not indicated. We assumed as both the doses were counted. Data on local any and systemic any adverse events could not be extracted (adverse events reported only singularly)
Clarck [12]	Oral temp. >38 °C	Mild: did not interfere with activity<br>Moderate: caused some impairment<br>Severe: affected daily activity and required medical attention	Any local reaction among: pain, bruising, erythema, and swelling	Any systemic reaction among: chills, malaise, muscle aches, nausea, and headache	7-day self-compiled diary card	Data on any local and systemic adverse events could not be extracted (adverse events reported only singularly)
Di [13]	NR	NR	NR	NR	NR	No data on adverse events have been reported
Esposito [14]	Axillary temp. ≥38 °C	Mild: did not interfere with activity<br>Moderate: caused some impairment<br>Severe: affected daily activity and required medical attention	Any local reaction among: erythema, swelling/induration, pain	Any systemic reaction among: irritability, sleepiness, changes in eating habits, vomiting, diarrhea, malaise, muscle ache	14-day self-compiled diary card	None
Gasparini 1,2 [55]	NR	NR	Any local reaction among: ecchymosis, induration, erythema, swelling, pain	Any systemic reaction among: chills, malaise, myalgia, arthralgia, headache, sweating, fatigue, nausea, fever	7-day self-compiled diary card	Data about any local and systemic adverse could not be extracted (adverse events reported only singularly)
Greenberg [15]	Oral temp.<br>None:<37.7 °C<br>Mild: ≥37.7 to <38 °C<br>Moderate: ≥38 to <39 °C<br>Severe: ≥39 °C	Mild: Did not interfere with activity<br>Moderate: Interfered with activity<br>Severe: Prevented daily activity	Any local reaction among: ecchymosis, induration, redness, tenderness, pain	Any systemic reaction among: chills, malaise, myalgia, headache, nausea, fever	7-day self-compiled diary	None

Igari [57]	Oral temp. None:<37.7 °C Mild: ≥37.7 to <38 °C Moderate: ≥38 to <39 °C Severe: ≥39 °C	Mild: Did not interfere with activity Moderate: Interfered with activity Severe: Prevented daily activity	Any local reaction among: ecchymosis, induration, redness, tenderness, pain	Any systemic reaction among: chills, malaise, myalgia, headache, nausea, fever	7-day interview form card	None
Ikematsu [16]	Four-points scale Grade 3: axillary temp. ≥39 °C Grade 4: axillary temp. ≥40 °C	Three points scale Grade 3: symptoms that hindered normal daily activities. The definitions of the other grades were not reported.	Any local reaction among: swelling, redness, pain	Any systemic reaction among: fatigue, headache, joint pain, muscle ache, shivering, sweating and fever	7-day self-compiled diary card	Data about any local and systemic adverse could not be extracted (adverse events reported only singularly)
Kung/Kao [17,18]	Axillary temp. ≥38.5 °C	NR	Any local reaction among: ecchymosis, swelling, redness, pain, decreased limb mobility	Any systemic reaction among: nasal congestion, cough, sore throat, headache, joint pain, muscle ache, nausea, vomiting, malaise, fever	7-day self-compiled diary card	None
Liang/Zhu/Wu [19,36,38]	Axillary temp. Mild: 37.1-37.5 °C Moderate: 37.6-39.0 °C Severe: >39.0 °C	Mild: Did not interfere with activity Moderate: Interfered with activity Severe: Prevented daily activity	Any local reaction among: swelling, redness, pain, induration	Any systemic reaction among: cough, headache, headache, myalgia, diarrhea, fatigue, fever	Diary cards, spontaneous reports and hospital admission records throughout the study	Data on specific adverse events are not reported separately by vaccine group and age group (and could thus only be included in non age-stratified meta-analyses)
Loebermann [20]	Axillary temp. ≥38 °C	Mild: Did not interfere with activity Moderate: Caused some impairment Severe: Affected daily activity and required medical attention	Any local reaction among: ecchymosis, erythema, induration, swelling, pain	Any systemic reaction among: chills/shivering, malaise, myalgia, arthralgia, headache, sweating, fatigue, fever	3-day self-compiled diary card	Most of the unsolicited adverse events are not reported. Data about fever have not been reported, and data on any local and systemic adverse could not be extracted (adverse events reported only singularly)
Lu [21]	Axillary temp. ≥38 °C	Mild: Did not interfere with activity Moderate: Caused some impairment Severe: Affected daily activity and required medical attention	Any local reaction among: soreness/pain, swelling, redness, ecchymosis and limitation of arm motion	Any systemic reaction among: nasal congestion, cough, sore throat, muscle aches, headache, vomiting, nausea and malaise	7-day self-compiled or compiled by parents diary card	None
Madhun [22]	Mild: 38.0-38.49 °C Moderate: 38.5-38.99 °C Severe: >39.0 °C	Mild: Did not interfere with activity Moderate: Caused some impairment Severe: Affected daily activity and required medical attention	Any local reaction among: erythema/redness, itching, edema/swelling, pain, ecchymosis, induration	Any systemic reaction among: fever, fatigue, headache, sweating, myalgia, diarrhea	7-day self-compiled diary card	None
Mallory [23]	Axillary temp. ≥38.3 °C	NR	NR	NR	Diary card	Data on pain are not reported. Data on any local adverse reaction could not be extracted (local adverse events reported only singularly)

Mironov 1,2 [24]	NR	NR	NR	NR	NR	The dosages of each arm have not been reported, thus data on adverse events could not be extracted
Nicholson [25]	Axillary temp. ≥38 °C	Mild: Did not interfere with activity<br>Moderate: Caused some impairment<br>Severe: Affected daily activity and required medical attention	Any local reaction among: pain, redness, swelling, bruising	Any systemic reaction among: muscle aches, chills, malaise, headache, nausea, fever	7-day self-compiled diary card	Data on adverse events are not reported separated by age groups and only for the second dose. Data on any local and systemic adverse reactions could not be extracted (adverse events reported only singularly)
Nolan [26]	Mild: ≥37.5 to ≤38.5<br>Moderate: >38.5 to ≤39.5<br>Severe: >39.5	Mild: Did not interfere with activity<br>Moderate: Caused some impairment<br>Severe: Affected daily activity and required medical attention	Any local reaction among: pain, redness, swelling	Any systemic reaction among: muscle aches, chills, malaise, headache, nausea, irritability, loss of appetite, diarrhea, fever	7-day diary compiled by parents	None
Oh [27]	Axillary temp. ≥38 °C	Mild: Did not interfere with activity<br>Moderate: Caused some impairment<br>Severe: Affected daily activity and required medical attention	Any local reaction among: pain, tenderness, redness and swelling	Any systemic reaction among: fever, headache, malaise, shivering, fatigue, sweating, myalgia and arthralgia (subjects ≥3 years of age) or fever, irritability, crying, loss of appetite, vomiting, diarrhea and drowsiness	7-day diary card compiled by parents	Data on any local and systemic adverse reactions could not be extracted (adverse events reported only singularly)
Plennevaux [28,29]	Grade 3: >39.5 for infants aged 6-23months, or>39.0 ◦C for children aged 2-9years. Definitions of the other grades were not reported	Three points scale<br>Grade3: incapacitating, preventing usual activities.<br>The definitions of the other grades were not reported.	Any local reaction among: tenderness, erythema, swelling, induration, ecchymosis	Any systemic reaction among: fever, vomiting, abnormal crying, drowsiness, lost of appetite, irritability	7-day diary compiled by parents	None
Roman 1 [30]	Axillary temp. ≥38 °C	No effect on normal activities: Pain on touching site<br>Some interference with normal everyday activities: pain on moving limb<br>Prevents normal everyday activities: significant pain at rest	Any local reaction among: pain, redness, swelling	Any systemic reaction among: fatigue, headache, arthralgia, myalgia, shivering, sweating, fever	7-day self-compiled diary card	Data on any local and systemic adverse reactions could not be extracted (adverse events reported only singularly)
Roman 2 [31]	Grade 1: 38.0-38.5 °C<br>Grade 2: 38.51-39.0 °C<br>Grade 3: >39.0 °C	Grade 1: easily tolerated, pain on touch<br>Grade 2: interferes with normal activity, when limb is moved<br>Grade 3: prevents normal activity, significant pain at rest	Any local reaction among: pain, redness, swelling	Any systemic reaction among: fatigue, headache, arthralgia, myalgia, shivering, sweating, fever	7-day self-compiled diary card	Data on any local and systemic adverse reactions could not be extracted (adverse events reported only singularly)
Roman 3 [39]	Axillary temp. ≥38 °C	NR	NR	NR	NR	The frequencies of fever, any local and systemic adverse reactions have not been reported

Scheifele [58]	Oral temp. ≥38.5 °C	Mild: Did not interfere with activity<br>Moderate: Caused some impairment<br>Severe: Affected daily activity and required medical attention	Any local reaction among: pain, redness, swelling	Any systemic reaction among: irritability, drowsiness, sleep disturbance, decreased appetite, fever	7-day diary card compiled by parents	None
Sun [32]	NR	NR	NR	NR	NR	No data on adverse events have been reported
Talaat [33]	NR	Mild: Did not interfere with activity<br>Moderate: Caused some impairment<br>Severe: Affected daily activity and required medical attention	Any local reaction among: pain, erythema, induration, tenderness, ecchymosis	Any systemic reaction among: headache, malaise, myalgia, nausea, chills, vomit, fever	7-day self-compiled diary card	Data on adverse events have not been reported separately by age group (they have been used only in non age-stratified meta-analyses)
Vajo [34]	Oral temp. >38 °C for ≥24h	Mild: transient or mild discomfort<br>Moderate: mild-to moderate limitation in activity<br>Severe: substantial limitation in activity	Any local reaction among: pain, erythema, induration, ecchymosis	Any systemic reaction among: headache, malaise, myalgia, shivering, fatigue, rash, fever	7-day self-compiled diary card	Data on adverse events were not reported separated by age groups
Waddington [35]	Axillary temp. ≥38 °C	Mild: Did not interfere with activity<br>Moderate: Caused some impairment<br>Severe: Affected daily activity and required medical attention	Any local reaction among: pain, redness, swelling	Any systemic reaction among: headache, malaise, myalgia, loss of appetite, fever	7-day diary card compiled by parents	Data on any systemic adverse reaction could not be extracted (systemic adverse events reported only singularly)
Yasuda [37]	Axillary temp. ≥38 °C	Mild: Did not interfere with activity<br>Moderate: Caused some impairment<br>Severe: Affected daily activity and required medical attention	Any local reaction among: ecchymosis, erythema, induration, swelling, and pain at injection site	Any systemic reaction among: headache, arthralgia, chills, fatigue, malaise, myalgia, nausea, sweating, and fever	7-day diary card compiled by parents	Data on any local and systemic adverse reactions could not be extracted (adverse events reported only singularly)

NR: Not reported.
