# Supplementary material for: Meta-Analysis of the Immunogenicity and Tolerability of Pandemic Influenza A 2009 (H1N1) Vaccines
Source: PLoS One. 2011 Sep 6;6(9):e24384. doi: 10.1371/journal.pone.0024384 (PMC3167852; doi:10.1371/journal.pone.0024384)
Supplement: Table S6 — Rates of injection-site pain, fever, any systemic and local adverse event after one or two doses of 2009 H1N1 influenza vaccination, according to vaccine dose and formulation (presence or absence of an adjuvant). Data from single studies have been combined using proportion meta-analysis (random-effect model). (PDF) [file pone.0024384.s006.pdf]

Table S6. Rates of injection-site pain, fever, any systemic and local adverse event after one or two doses of 2009 H1N1 influenza vaccination, according to vaccine dose and formulation (presence or absence of an adjuvant). Data from single studies have been combined using proportion meta-analysis (random-effect model).

	**Pain**			**Fever**			**Any systemic adverse event**			**Any local adverse event**		
Formulations*	% (95%CI)	N	References	% (95%CI)	N	References	% (95%CI)	N	References	% (95%CI)	N	References
Non-adjuvanted												
7.5x1 – All	20 (10-32)	985	[12,21,27,33,35]	4 (2-8)	985	[12,21,27,33,35]	32 (27-38)	921	[21,28,29,33]	21 (5-44)	1378	[21,28,29,33,35]
- Split-virus	17 (1-29)	525	[12,21,27,33]	2 (1-4)	525	[12,21,27,33]	32 (27-38)	921	[21,28,29,33]	30 (20-42)	918	[21,28,29,33]
- Whole-virus	27 (22-32)	460		7 (5-10)	460		--	0		1 (0-1)	460	[35]
7.5x2 – All	26 (15-39)	1301	[12,21,25,27,28,33,35]	5 (3-8)	1362	[12,21,25,27,28,33,35]	28 (13-47)	1606	[19,21,28,33]	16 (1-44)	2052	[19,21,28,33,35]
- Split-virus	25 (1-49)	683	[12,21,27,28,33]	5 (2-9)	744	[12,21,27,28,33]	28 (13-47)	1606	[19,21,28,33]	23 (1-62)	1606	[19,21,28,33]
- Whole-virus	28 (25-32)	618		6 (1-14)	618		--	0		1 (0-2)	460	[35]
15x1 (all split-virus)	30 (18-44)	1883	[8,12,17,18,20,21,26,27,33,54,57]	3 (0-8)	1678	[8,12,17,18,21,26,27,33,54,57]	31 (23-41)	2334	[8,17,18,19,21,26,28,29,33,38,54,57]	31 (19-45)	2332	[8,17,18,19,21,26,28,29,33,38,54,57]
15x2 (all split-virus)	32 (23-42)	2238	[8,11,12,15,17,18,21,26,27,28,33,38,54]	5 (2-10)	2060	[11,12,15,17,18,21,26,27,28,33,38]	37 (26-49)	5852	[8,15,17,18,19,21,26,28,33,54]	39 (19-61)	5852	[8,15,17,18,19,21,26,28,33,54]
21-30x1 (all split-virus)	42 (27-58)	880	[8,17,18,26,30,33,54]	7 (0-22)	880	[8,17,18,26,30,33,54]	35 (22-50)	1497	[8,17,18,26,29,33,38,54]	36 (18-56)	1497	[8,17,18,26,29,33,38,54]
21-30x2 (all split-virus)	32 (23-42)	1610	[8,11,15,17,18,19,26,33,38,54]	8 (0-20)	1501	[11,15,17,18,19,26,33,38]	42 (27-58)	4574	[11,15,17,18,19,26,33]	43 (19-68)	4574	[8,15,17,18,19,26,33,54]
Adjuvanted												
1.88-5.25x1 – All	63 (44-79)	1899	[9,12,22,30,31,34,35,37,55,58]	4 (1-9)	1780	[9,12,22,30,31,34,35,37,55,58]	51 (15-86)	869	[9,22,34,58]	37 (10-71)	1320	[9,22,34,35,58]
- Aluminum	7 (4-10)$	355	[34]	0 (0-1)$	355	[34]	8 (5-11)$	355	[34]	13 (10-17)$	355	[34]
- Other adjuvants	68 (57-78)	1544		5 (2-10)	1425		67 (57-76)	514		45 (8-86)	965	
1.88-5.25x2 – All	66 (56-75)	1248	[9,12,25,31,35,37,58]	12 (2-29)	1129	[9,12,25,31,35,37,58]	61 (15-97)	402	[9,19,58]	29 (5-61)	832	[9,19,35,58]
- Aluminum	--	0		--	0		12 (6-20)$	101	[19]	9 (4-16)$	101	[19]
- Other adjuvants	66 (56-75)	1248	[9,12,25,31,35,37,58]	12 (2-29)	1129	[9,12,25,31,35,37,58]	84 (74-92)	301		36 (5-77)	731	
7.5x1 – All	52 (31-72)	565	[8,12,14,37,54,55]	3 (0-8)	576	[8,12,14,37,54,55]	19 (3-43)	582	[8,14,38,54]	28 (3-65)	583	[8,14,38,54]

- Aluminum	--	0		--	0		1 (7-12)	440	[38]	10 (7-13)	440	[38]
- Other adjuvants	52 (31-72)	565	[8,12,14,37,54,55]	3 (0-8)	576	[8,12,14,37,54,55]	25 (4-56)	142		40 (11-74)	143	
7.5x2 – All	59 (27-87)	624	[8,12,37,38,54]	9 (2-19)	578	[12,37,38]	25 (5-53)	850	[8,19,54]	36 (6-75)	850	[8,19,54]
- Aluminum	22 (18-26)	440	[38]	15 (11-18)	440	[38]	14 (11-17)	743	[19]	18 (16-21)	743	[19]
- Other adjuvants	71 (59-82)	184		5 (0-22)	88		39 (30-49)	107		57 (47-67)	107	
15x1***	84 (64-95)	25	[12]	4 (0-20)	576	[12]	11 (8-14)	440	[38]	12 (9-16)	440	[38]
15x2****	20 (16-24)	440	[38]	15 (12-18)	578	[38]	14 (11-17)	741	[19]	17 (14-20)	741	[19]

N = total number of subject analyzed; CI = Confidence Intervals. * 7.5x1 = Results collected after the first or single dose of 7.5 mcgs of hemagglutin antigen; 7.5x2 = Results collected after the second dose of 7.5 mcgs of hemagglutinin antigen. *** Other adjuvants only. **** Aluminum only. $^{\$}$Whole-virus only.
