# Supplementary material for: Meta-Analysis of the Immunogenicity and Tolerability of Pandemic Influenza A 2009 (H1N1) Vaccines
Source: PLoS One. 2011 Sep 6;6(9):e24384. doi: 10.1371/journal.pone.0024384 (PMC3167852; doi:10.1371/journal.pone.0024384)
Supplement: Table S7 — Direct meta-analyses comparing the rates of pain at the injection site of H1N1 influenza vaccines at higher versus next lower doses of hemagglutinin antigen (HA) and of adjuvanted versus non-adjuvanted H1N1 influenza vaccines. All doses are reported as µg of HA. (PDF) [file pone.0024384.s007.pdf]

Table S7. Direct meta-analyses comparing the rates of pain at the injection site of H1N1 influenza vaccines at higher versus next lower doses of hemagglutinin antigen (HA) and of adjuvanted versus non-adjuvanted H1N1 influenza vaccines. All doses are reported as µg of HA.

All ages	RR (95%CI)	N	References
Non-adjuvanted vaccines			
15 vs 7.5 - One dose only	1.19 (0.82-1.71)	858	[12,33]
30 vs 15 - One dose only	1.14 (0.87-1.49)	1743	[8,18,26,33,54]
All dosages * - One dose only	1.10 (0.91-1.32)	2200	[8,12,18,26,33,54]
15 vs 7.5 - Two doses	0.99 (0.83-1.18)	1164	[12,28,33]
30 vs 15 - Two doses	1.20 (0.98-1.47)	3274	[8,11,15,18,26,33,38,54]
All dosages * - Two doses	1.13 (0.98-1.30)	4037	[8,11,12,15,18,26,28,33,38,54]
Adjuvanted vaccines			
7.5 vs 1.88-5.25 - One dose only	1.17 (1.00-1.38)	366	[12,37,55]
15 vs 7.5 - One dose only	1.15 (0.86-1.54)	51	[12]
All dosages * - One dose only	1.15 (0.99-1.33)	391	[12,37,55]
7.5 vs 1.88-5.25 - Two doses	1.27 (1.02-1.57)	152	[12,37]
15 vs 7.5 - Two doses	0.90 (0.70-1.16)	880	[38]
All dosages * - Two doses	1.01 (0.75-1.37)	1362	[12,37,38]
Adjuvanted vs non-adjuvanted			
7.5 - One dose only	2.03 (1.15-3.60)	51	[12]
15 - One dose only	2.10 (1.26-3.50)	50	[12]
All dosages * - One dose only	2.58 (1.71-3.87)	232	[12,39]
7.5 – Two doses	2.03 (1.15-3.60)	51	[12]
15 - Two doses	1.83 (1.32-2.54)	880	[38]
All dosages * - Two doses	1.79 (0.92-3.48)	1832	[12,38,39]
Stratification by adjuvant type			
Same dosage - One dose only *			
Aluminum	--	0	
MF-59	2.07 (1.41-3.03)	101	[12]
$AS03_{(A\ or\ B)}$	3.68 (2.38-5.69)	131	[39]
Same dosage - Two doses *			
Aluminum	1.19 (0.51-2.77)	1650	[38]
MF-59	2.03 (1.15-3.60)	51	[12]
$AS03_{(A\ or\ B)}$	3.68 (2.38-5.69)	131	[39]

N = total number of subject analyzed; RR= Random-effect risk ratio; CI = Confidence Intervals. * The total sample of overall meta-analyses may be lower (higher) than the sum of stratified meta-analyses because some arms had to be split to be included in more than a stratified meta-analysis (some arms were different from the main comparisons shown in the Table).
