# Supplementary material for: Meta-Analysis of the Immunogenicity and Tolerability of Pandemic Influenza A 2009 (H1N1) Vaccines
Source: PLoS One. 2011 Sep 6;6(9):e24384. doi: 10.1371/journal.pone.0024384 (PMC3167852; doi:10.1371/journal.pone.0024384)
Supplement: Table S8 — Direct meta-analyses comparing the rates of fever of H1N1 influenza vaccines at higher versus next lower doses of hemagglutinin antigen (HA) and of adjuvanted versus non-adjuvanted H1N1 influenza vaccines. All doses are reported as µg of HA. (PDF) [file pone.0024384.s008.pdf]

Table S8. Direct meta-analyses comparing the rates of <u>fever</u> of H1N1 influenza vaccines at higher versus next lower doses of hemagglutinin antigen (HA) and of adjuvanted versus non-adjuvanted H1N1 influenza vaccines. All doses are reported as µg of HA.

All ages	RR (95%CI)	N	(References)
Non-adjuvanted vaccines			
15 vs 7.5 - One dose only	0.89 (0.33-2.43)	858	[12,33]
30 vs 15 - One dose only	1.71 (1.28-2.28)	1743	[8,18,26,33,54]
All dosages * - One dose only	1.65 (1.24-2.19)	2200	[8,12,18,26,33,54]
15 vs 7.5 - Two doses	0.89 (0.51-1.56)	1281	[12,28,33]
30 vs 15 - Two doses	1.40 (1.14-1.72)	2997	[11,15,18,26,33,38]
All dosages * - Two doses	1.32 (1.09-1.61)	3877	[11,12,15,18,26,28,33,38]
Adjuvanted vaccines			
7.5 vs 1.88-5.25 - One dose only	1.35 (0.45-4.04)	387	[12,37,55]
15 vs 7.5 - One dose only	3.12 (0.13-73.06)	51	[12]
All dosages * - One dose only	1.38 (0.49-3.87)	512	[12,37,55]
7.5 vs 1.88-5.25 - Two doses	0.97 (0.36-2.59)	173	[12,37]
15 vs 7.5 - Two doses	1.06 (0.78-1.46)	880	[38]
All dosages * - Two doses	0.91 (0.69-1.20)	1383	[12,37,38]
Adjuvanted vs non-adjuvanted			
7.5 - One dose only	--	0	
15 - One dose only	3.00 (0.13-70.30)	50	[12]
All dosages * - One dose only	3.00 (0.13-70.30)	101	[12]
7.5 – Two doses	0.32 (0.01-7.53)	51	[12]
15 - Two doses	1.05 (0.76-1.43)	880	[38]
All dosages * - Two doses	0.81 (0.53-1.25)	1701	[12,38]
Stratification by adjuvant type			
Same dosage - One dose only *			
Aluminum	--	0	
MF-59	3.00 (0.13-70.30)	101	[12]
$AS03_{(A\ or\ B)}$	--	0	
Same dosage - Two doses *			
Aluminum	0.83 (0.51-1.33)	1650	[38]
MF-59	0.32 (0.01-7.53)	51	[12]
$AS03_{(A\ or\ B)}$	--	0	

N = total number of subject analyzed; RR= Random-effect risk ratio; CI = Confidence Intervals. * The total sample of overall meta-analyses may be lower (higher) than the sum of stratified meta-analyses because some arms had to be split to be included in more than a stratified meta-analysis (some arms were different from the main comparisons shown in the Table).
