# Supplementary material for: Meta-Analysis of the Immunogenicity and Tolerability of Pandemic Influenza A 2009 (H1N1) Vaccines
Source: PLoS One. 2011 Sep 6;6(9):e24384. doi: 10.1371/journal.pone.0024384 (PMC3167852; doi:10.1371/journal.pone.0024384)
Supplement: Table S9 — Direct meta-analyses comparing the rates of any systemic adverse event of H1N1 influenza vaccines at higher versus next lower doses of hemagglutinin antigen (HA) and of adjuvanted versus non-adjuvanted H1N1 influenza vaccines. All doses are reported as µg of HA. (PDF) [file pone.0024384.s009.pdf]

Table S9. Direct meta-analyses comparing the rates of <u>any systemic adverse event</u> of H1N1 influenza vaccines at higher versus next lower doses of hemagglutinin antigen (HA) and of adjuvanted versus non-adjuvanted H1N1 influenza vaccines. All doses are reported as µg of HA.

All ages	RR (95%CI)	N	(References)
Non-adjuvanted vaccines			
15 vs 7.5 - One dose only	1.00 (0.87-1.16)	1718	[28,29,33]
30 vs 15 - One dose only	1.08 (0.90-1.29)	3115	[8,18,26,29,33,38]
All dosages * - One dose only	1.06 (0.93-1.21)	4183	[8,18,26,28,29,33,38,54]
15 vs 7.5 - Two doses	1.22 (0.85-1.75)	6693	[19,28,33]
30 vs 15 - Two doses	1.22 (1.07-1.38)	10100	[8,15,18,19,26,33,54]
All dosages * - Two doses	1.22 (1.07-1.39)	11852	[8,15,18,19,26,28,33,54]
Adjuvanted vaccines			
7.5 vs 1.88-5.25 - One dose only	--	0	
15 vs 7.5 - One dose only	1.15 (0.77-1.72)	880	[38]
All dosages * - One dose only	1.02 (0.72-1.46)	1210	[38]
7.5 vs 1.88-5.25 - Two doses	1.17 (0.67-2.04)	844	[19]
15 vs 7.5 - Two doses	1.00 (0.78-1.29)	1484	[19]
All dosages * - Two doses	1.08 (0.85-1.36)	1915	[19]
Adjuvanted vs non-adjuvanted			
7.5 - One dose only	--	0	
15 - One dose only	0.87 (0.60-1.26)	880	[38]
All dosages * - One dose only	0.72 (0.49-1.05)	1650	[38]
7.5 – Two doses	1.36 (1.05-1.77)	1665	[19]
15 - Two doses	0.81 (0.67-0.97)	5281	[19]
All dosages * - Two doses	0.93 (0.72-1.21)	11157	[15,19]
Stratification by adjuvant type			
Same dosage - One dose only *			
Aluminum	0.72 (0.49-1.05)	1650	[38]
MF-59	--	0	
$AS03_{(A\ or\ B)}$	--	0	
Same dosage - Two doses *			
Aluminum	0.91 (0.73-1.13)	11157	[15,19]
MF-59	--	0	
$AS03_{(A\ or\ B)}$	--	0	

N = total number of subject analyzed; RR= Random-effect risk ratio; CI = Confidence Intervals. * The total sample of overall meta-analyses may be lower (higher) than the sum of stratified meta-analyses because some arms had to be split to be included in more than a stratified meta-analysis (some arms were different from the main comparisons shown in the Table).
