# Supplementary material for: Meta-Analysis of the Immunogenicity and Tolerability of Pandemic Influenza A 2009 (H1N1) Vaccines
Source: PLoS One. 2011 Sep 6;6(9):e24384. doi: 10.1371/journal.pone.0024384 (PMC3167852; doi:10.1371/journal.pone.0024384)
Supplement: Table S10 — Direct meta-analyses comparing the rates of any local adverse event of H1N1 influenza vaccines at higher versus next lower doses of hemagglutinin antigen (HA) and of adjuvanted versus non-adjuvanted H1N1 influenza vaccines. All doses are reported as µg of HA. (PDF) [file pone.0024384.s010.pdf]

Table S10. Direct meta-analyses comparing the rates of <u>any local adverse event</u> of H1N1 influenza vaccines at higher versus next lower doses of hemagglutinin antigen (HA) and of adjuvanted versus non-adjuvanted H1N1 influenza vaccines. All doses are reported as µg of HA.

All ages	RR (95%CI)	N	(References)
Non-adjuvanted vaccines			
15 vs 7.5 - One dose only	1.00 (0.71-1.40)	1713	[28,29,33]
30 vs 15 - One dose only	1.09 (0.96-1.23)	3113	[8,18,26,29,33,38,54]
All dosages * - One dose only	1.05 (0.93-1.19)	4178	[8,18,26,28,29,33,38,54]
15 vs 7.5 - Two doses	1.59 (0.72-3.54)	6693	[19,28,33]
30 vs 15 - Two doses	1.21 (1.03-1.42)	10100	[8,15,18,19,26,33,54]
All dosages * - Two doses	1.20 (0.96-1.51)	11576	[15,18,19,26,28,33]
Adjuvanted vaccines			
7.5 vs 1.88-5.25 - One dose only	--	0	
15 vs 7.5 - One dose only	1.29 (0.88-1.88)	880	[38]
All dosages * - One dose only	1.31 (0.96-1.79)	1210	[38]
7.5 vs 1.88-5.25 - Two doses	2.05 (1.08-3.90)	884	[19]
15 vs 7.5 - Two doses	0.93 (0.75-1.16)	1484	[19]
All dosages * - Two doses	1.45 (0.87-2.42)	1915	[19]
Adjuvanted vs non-adjuvanted			
7.5 - One dose only	--	0	
15 - One dose only	2.08 (1.33-3.25)	880	[38]
All dosages * - One dose only	2.49 (1.74-3.57)	1650	[38]
7.5 – Two doses	169 (23.7-1204)	1665	[19]
15 - Two doses	2.91 (2.39-3.55)	5281	[19]
All dosages * - Two doses	4.91 (2.35-10.25)	10917	[19]
Stratification by adjuvant type			
Same dosage - One dose only *			
Aluminum	2.49 (1.74-3.57)	1650	[38]
MF-59	--	0	
$AS03_{(A\ or\ B)}$	--	0	
Same dosage - Two doses *			
Aluminum	4.91 (2.35-10.25)	10917	[19]
MF-59	--	0	
$AS03_{(A\ or\ B)}$	--	0	

N = total number of subject analyzed; RR= Random-effect risk ratio; CI = Confidence Intervals. * The total sample of overall meta-analyses may be lower (higher) than the sum of stratified meta-analyses because some arms had to be split to be included in more than a stratified meta-analysis (some arms were different from the main comparisons shown in the Table).
