# Supplementary material for: Meta-Analysis of the Immunogenicity and Tolerability of Pandemic Influenza A 2009 (H1N1) Vaccines
Source: PLoS One. 2011 Sep 6;6(9):e24384. doi: 10.1371/journal.pone.0024384 (PMC3167852; doi:10.1371/journal.pone.0024384)
Supplement: Table S11 — Absolute percentages of subjects who reported one adverse event (injection-site pain, fever, any systemic or any local) after the second dose of 2009 H1N1 vaccine, and who did not report such event after the first dose (according to vaccine dose and formulation – presence or absence of an adjuvant). Data from single studies have been combined using proportion meta-analysis (random-effect model). All doses are reported as µg of hemagglutinin antigen. (PDF) [file pone.0024384.s011.pdf]

Table S11. Absolute percentages of subjects who reported one adverse event (injection-site pain, fever, any systemic or any local) after the second dose of 2009 H1N1 vaccine, and who did not report such event after the first dose (according to vaccine dose and formulation – presence or absence of an adjuvant). Data from single studies have been combined using <u>proportion meta-analysis</u> (random-effect model). All doses are reported as µg of hemagglutinin antigen.

	**Pain**			**Fever**			**System. Any**			**Local Any**		
Formulations*	% (95%CI)	N	References	% (95%CI)	N	References	% (95%CI)	N	References	% (95%CI)	N	References
<u>Non-adjuvanted</u>												
7.5x2 vs 7.5x1 (All split virus)	1 (0-2)	971	[12,21,27,33,35]	1 (0-4)	971	[12,21,27,33,35]	2 (1-14)	684	[21,28,33]	3 (0-11)	1130	[21,28,33,35]
15x2 vs 15x1 (All split-virus)	0 (0-2)	1227	[8,12,18,21,26,27,33,54]	0 (0-1)	1059	[12,18,21,26,27,33]	1 (0-4)	1192	[8,18,21,26,28,33,54]	2 (0-7)	1192	[8,18,21,26,28,33,54]
30x2 vs 30x1 (All split-virus)	0 (0-1)	813	[8,18,26,33,54]	0 (0-1)	704	[18,26,33]	0 (0-1)	813	[8,18,26,33,54]	0 (0-1)	813	[8,18,26,33,54]
<u>Adjuvanted</u>												
1.88-5.25x2 vs 1.88-5.25x1 (All split virus)	2 (0-4)	1073	[9,12,31,35,37,39,58]	7 (1-21)	954	[9,12,31,35,37,58]	12 (0-37)	301	[9,58]	3 (2-5)	731	[9,35,58]
7.5x2 vs 7.5x1 (All split virus)	0 (0-1)	184	[8,12,37,54]	1 (0-3)	138	[12,37]	--	0		0 (0-3)	107	[8,54]

N = total number of subject analyzed; (ref) = References to included studies; CI = Confidence Intervals. * 7.5x1 = Results collected after the first or single dose of 7.5 µg; 7.5x2 = Results collected after the second dose of 7.5 µg. ** Participants received a vaccine with less than 4 mcgs of hemagglutinin antigen in all trials.
