# Supplementary material for: Meta-Analysis of the Immunogenicity and Tolerability of Pandemic Influenza A 2009 (H1N1) Vaccines
Source: PLoS One. 2011 Sep 6;6(9):e24384. doi: 10.1371/journal.pone.0024384 (PMC3167852; doi:10.1371/journal.pone.0024384)
Supplement: Table S12 — Rates of seroconversion after H1N1 influenza vaccination, according to vaccine dose (in µg of hemagglutinin antigen), formulation (presence or absence of an adjuvant; one administration or two), and methodological quality (lower or higher risk of bias, see text for details). Data from single studies have been combined using proportion meta-analysis (random-effect model). Non randomized and randomized trials were included. (PDF) [file pone.0024384.s012.pdf]

Table S12. Rates of seroconversion * after H1N1 influenza vaccination, according to vaccine dose (in μg of hemagglutinin antigen), formulation (presence or absence of an adjuvant; one administration or two), and methodological quality (lower or higher risk of bias, see text for details). Data from single studies have been combined using proportion meta-analysis (random-effect model). Non randomized and randomized trials were included.

	Adults [1]		Elderly [2]		Adolescents [3]		Children [4]		All ages
Formulations**	% (95%CI)	N (ref)	% (95%CI)	N (ref)	% (95%CI)	N (ref)	% (95%CI)	N (ref)	% (95%CI)
**Non-adjuvanted**									
7.5x1									
- Lower risk of bias	81 (77-85)	600 [19,25,28,29]	67 (40-88)	276 [19,25,28,29]	96 (93-98)	218 [19]	44 (21-69)	[19,21,27,28,29]	51 (29-72)
- Higher risk of bias	79 (51-96)	320 [12,13,33]	51 (44-58)	201 [33]	--	0	--	0	73 (61-84)
7.5x2									
- Lower risk of bias	80 (26-99)	442 [19,25]	64 (11-100)	170 [19,25]	99 (96-100)	195 [19]	83 (68-94)	915 [19,21,27,28,35]	77 (59-92)
- Higher risk of bias	78 (56-93)	23 [12]	--	0	--	0	--	0	78 (56-93)
15x1									
- Lower risk of bias	82 (63-96)	2247 [15,18,19,20,28,29,32,39,57]	81 (74-87)	1058 [17,19,20,28,29]	87 (68-98)	1251 [19,21,27]	62 (42-79)	1671 [19,21,26,27,28,29]	75 (63-86)
- Higher risk of bias	80 (65-92)	336 [11,12,33]	58 (53-64)	309 [11,33]	96 (90-99)	83 [8,54]	72 (61-82)	83 [8,54]	74 (66-81)
15x2									
- Lower risk of bias	91 (83-97)	1367 [15,18,19,39]	86 (54-100)	763 [18,19]	92 (75-100)	1074 [19,21,27]	91 (79-98)	1481 [19,21,26,27,28,29]	90 (82-96)
- Higher risk of bias	84 (68-95)	137 [11,12]	59 (49-68)	109 [11]	91 (82-96)	77 [8,54]	97 (94-99)	75 [8,54]	80 (68-89)
21-30x1									
- Lower risk of bias	92 (87-96)	1377 [15,18,19,28,29,30]	89 (84-93)	830 [17,19,28,29]	98 (96-99)	863 [19]	91 (83-96)	1058 [19,26]	91 (88-94)
- Higher risk of bias	94 (92-97)	315 [11,33]	74 (69-78)	313 [11,33]	100 (94-100)	57 [8,54]	75 (62-86)	53 [8,54]	85 (82-87)
21-30x2									
- Lower risk of bias	94 (90-98)	1032 [15,18,19,30]	91 (68-100)	605 [18,19]	99 (99-100)	717 [19]	98 (97-99)	921 [19,26]	95 (89-98)
- Higher risk of bias	94 (88-98)	115 [11]	78 (70-86)	111 [11]	92 (81-98)	52 [8,54]	92 (80-98)	48 [8,54]	89 (83-93)
**Adjuvanted**									
- Aluminum (any dose)		All trials at lower risk of bias							
- Other adjuvants									
1.88-5.25x1									

- Lower risk of bias	92 (85-97)	520 [22,25,30,39]	51 (34-68)	37 [25]	--	0	--	0	91 (82-97)
- Higher risk of bias	93 (90-95)	312 [12,16,31,55]	79 /71-86)	119 [31]	62 (42-79)	29 [37]	82 (55-98)	329 [9,37,58]	87 (78-94)
1.88-5.25x2									
- Lower risk of bias	98 (92-100)	308 [25,30,39]	73 (56-86)	37 [25]	--	0	99 (97-100)	392 [35]	98 (92-100)
- Higher risk of bias	98 (92-100)	191 [12,16,31]	94 (85-98)	67 [31]	100 (88-100)	29 [37]	99 (98-100)	321 [9,37,58]	99 (97-100)
7.5x1									
- Lower risk of bias	--	0	--	0	100 (90-100)	36 [14]	--	0	100 (90-100)
- Higher risk of bias	84 (56-99)	242 [12,19,55]	73 (65-80)	154 [19,55]	90 (65-100)	81 [8,37,54]	85 (67-97)	84 [8,19,37]	82 (69-92)
7.5x2									
- Lower risk of bias	--	0	--	0	--	0	--	0	--
- Higher risk of bias	92 (73-99)	24 [12,19]	--	0	93 (70-100)	74 [8,19,37]	97 (91-100)	76 [8,19,37,54]	95 (84-100)
15x1									
- Lower risk of bias	--	0	--	0	--	0	--	0	--
- Higher risk of bias	88 (69-97)	25 [12]	--	0	--	0	--	0	88 (69-97)
15x2 (no trials; all Aluminum)									

N = total number of subject analyzed; (ref) = References to included studies; CI = Confidence Intervals; [1] Adults = from 18 to 64 years; [2] Elderly = from 65 years; [3] Adolescents = from 10 to 17 years; [4] Children = from 6 months to 9 years (see Table S1 for several exceptions). * Seroconversion = subjects with a pre-vaccination hemagglutinination-inhibition antibody titer <=1:10 and a post-vaccination titer >=1:40, or a pre-vaccination titer >=1:10 and an increase in the titer by a factor of four or more after vaccination. ** 7.5x1 = Results collected after the first or single dose of 7.5µg; 7.5x2 = Results collected after the second dose of 7.5µg.
